# Estimated Sustainable Cost-Based Prices for Diabetes Medicines

**DOI:** 10.1001/jamanetworkopen.2024.3474

**Published:** 2024-03-27

**Authors:** Melissa J. Barber, Dzintars Gotham, Helen Bygrave, Christa Cepuch

**Affiliations:** 1 Yale Collaboration for Regulatory Rigor, Integrity, and Transparency (CRRIT), New Haven, Connecticut; 2 Program on Regulation, Therapeutics, and Law, Division of Pharmacoepidemiology and Pharmacoeconomics, Department of Medicine, Brigham and Women’s Hospital, and Harvard Medical School, Boston, Massachusetts; 3 Department of Global Health and Population, Harvard T.H. Chan School of Public Health, Boston, Massachusetts; 4 King’s College Hospital, London, United Kingdom; 5 Médecins Sans Frontières Access Campaign, Geneva, Switzerland

## Abstract

**Importance:**

The burden of diabetes is growing worldwide. The costs associated with diabetes put substantial pressure on patients and health budgets, especially in low- and middle-income countries. The prices of diabetes medicines are a key determinant for access, yet little is known about the association between manufacturing costs and current market prices.

**Objectives:**

To estimate the cost of manufacturing insulins, sodium-glucose cotransporter 2 inhibitors (SGLT2Is), and glucagonlike peptide 1 agonists (GLP1As), derive sustainable cost-based prices (CBPs), and compare these with current market prices.

**Design, Setting, and Participants:**

In this economic evaluation, the cost of manufacturing insulins, SGLT2Is, and GLP1As was modeled. Active pharmaceutical ingredient cost per unit (weighted least-squares regression model using data from a commercial database of trade shipments, data from January 1, 2016, to March 31, 2023) was combined with costs of formulation and other operating expenses, plus a profit margin with an allowance for tax, to estimate CBPs. Cost-based prices were compared with current prices in 12 countries, collected in January 2023 from public databases. Countries were selected to provide representation of different income levels and geographic regions based on the availability of public databases.

**Main Outcomes and Measures:**

Estimated CBPs; lowest current market prices (2023 US dollars).

**Results:**

In this economic evaluation of manufacturing costs, estimated CBPs for treatment with insulin in a reusable pen device could be as low as $96 (human insulin) or $111 (insulin analogues) per year for a basal-bolus regimen, $61 per year using twice-daily injections of mixed human insulin, and $50 (human insulin) or $72 (insulin analogues) per year for a once-daily basal insulin injection (for type 2 diabetes), including the cost of injection devices and needles. Cost-based prices ranged from $1.30 to $3.45 per month for SGLT2Is (except canagliflozin: $25.00-$46.79) and from $0.75 to $72.49 per month for GLP1As. These CBPs were substantially lower than current prices in the 12 countries surveyed.

**Conclusions and Relevance:**

High prices limit access to newer diabetes medicines in many countries. The findings of this study suggest that robust generic and biosimilar competition could reduce prices to more affordable levels and enable expansion of diabetes treatment globally.

## Key Points

**Question:**

What could prices of insulins, sodium-glucose cotransporter 2 inhibitors (SGLT2Is), and glucagonlike peptide 1 agonists (GLP1As) be if they were closer to the cost of production?

**Findings:**

In this economic evaluation of manufacturing costs, estimated cost-based prices per month were US $1.30 to $3.45 for SGLT2Is (except canagliflozin), and $0.75 to $72.49 for GLP1As, substantially lower than current market prices in nearly all comparisons. Twice-daily mixed human insulin NPH could cost $61 per year, while basal-bolus treatment with insulin glargine and aspart could cost $111 per year, with reusable pen formulations having the lowest estimated prices.

**Meaning:**

The findings of this study suggest that insulins, SGLT2Is, and GLP1As can likely be manufactured for prices far below current prices, enabling wider access.

## Teaser

This economic evaluation examines the health expenditures related to medicines used for treatment of diabetes worldwide.

## Introduction

While the burden of diabetes is increasing worldwide, health systems are faced with unaffordable medicine prices. There were an estimated 537 million people living with diabetes (PLD) worldwide in 2021, 90% of whom live in low- and middle-income countries (LMICs).^1^ Health expenditures directly related to diabetes have tripled in the past 15 years.^1^ Major challenges remain in accessing insulin and newer treatments for type 2 diabetes (T2D).^2,3,4,5,6,7,8,9^

Insulin analogues offer different pharmacokinetic profiles that allow insulin needs to be matched more closely, enable more convenient dosing regimens, and, in some cases, reduce the rate of adverse events.^10^ Newer treatments for T2D—sodium-glucose cotransporter 2 inhibitors (SGLT2Is) and glucagonlike peptide 1 agonists (GLP1As)—are now recommended for first-line treatment of T2D for patients with additional cardiovascular risk factors or obesity, independent of metformin use.^11^

Understanding the cost of manufacture can support health systems to target a reasonable price during negotiations with pharmaceutical manufacturers. Earlier analyses by some of the authors of the study reported herein estimated the cost of manufacturing certain diabetes medicines, including insulins,^12,13,14^ finding that estimated cost-based prices were far below the market prices for insulin analogues at the time. Manufacturing cost estimates for some GLP1As (semaglutide and liraglutide) were recently published in the context of obesity treatment.^15^ This study develops methods for estimating pharmaceutical manufacturing costs, updates cost analyses for insulins,^12^ and provides, to our knowledge, the first published manufacturing cost estimates for SGLT2Is and GLP1As for the treatment of diabetes.

## Methods

This economic evaluation study estimated the cost of production for insulins, SGLT2Is, and GLP1As, and, based on this, a sustainable cost-based price (CBP), and compared CBPs with the current lowest reported prices in 12 countries, collected in January 2023 from public databases. Cost-based prices were defined as prices that would be expected in competitive markets that afford manufacturers sustainable returns, while avoiding excessive profit margins. The protocol was submitted to the institutional review board at the Harvard T.H. Chan School of Public Health, which determined that this research was not human research as defined by Department of Health and Human Service regulations 45 CFR 46.102(e) or the US Food and Drug Administration (FDA) regulations. This study followed the relevant portions of the Consolidated Health Economic Evaluation Reporting Standards (CHEERS) reporting guideline.

We included all SGLT2Is, GLP1As, and insulins approved by the FDA or European Medicines Agency in all available formulations. We did not include combination products, except for 70/30 mixed human insulin NPH, which was included due to its widespread use.

**Figure 1.**
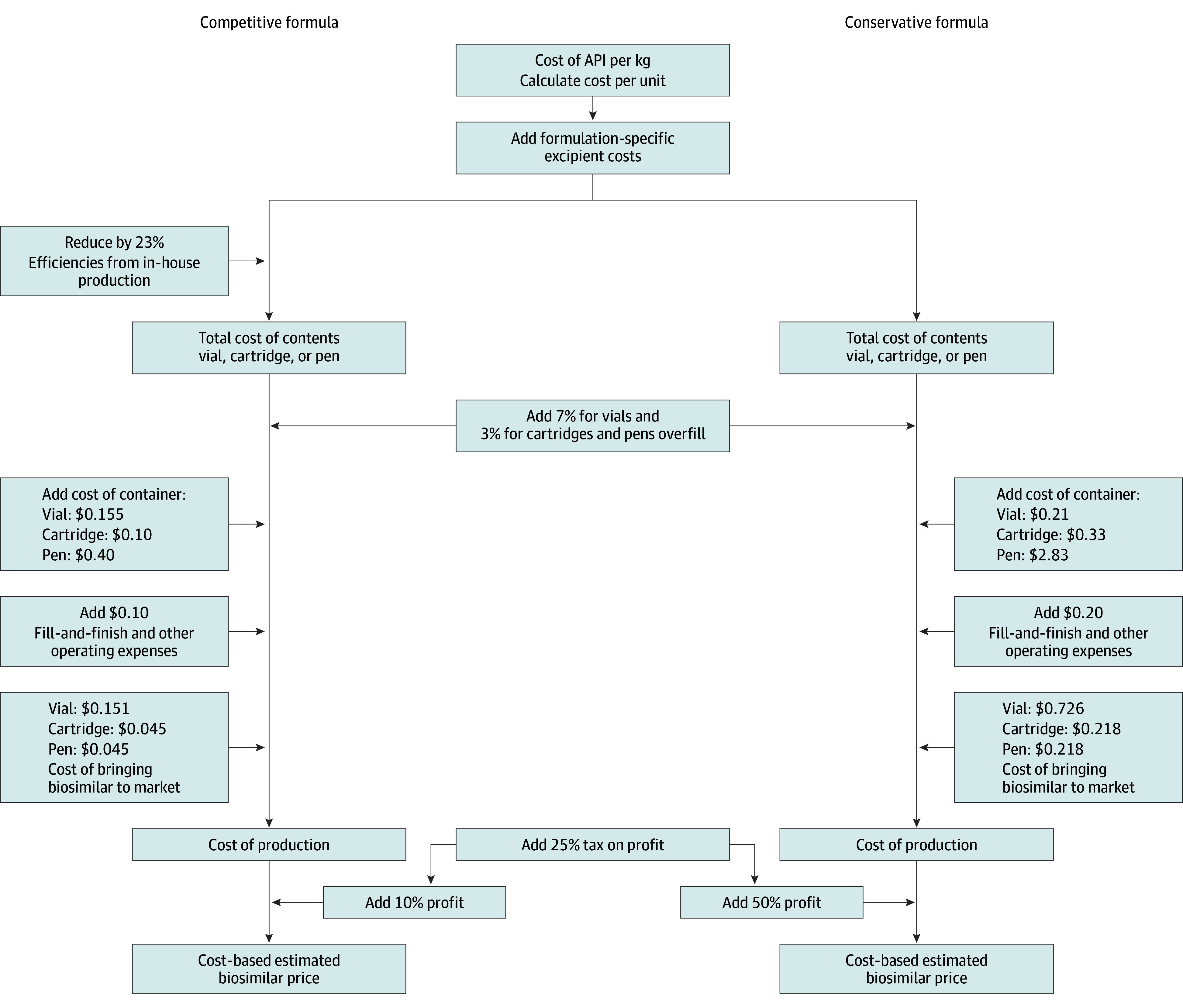
Cost-Based Estimated Price Algorithm for Insulins and Injectable and Glucagonlike Peptide 1 Agonists (GLP1As) API indicates active pharmaceutical ingredient.

**Figure 2.**
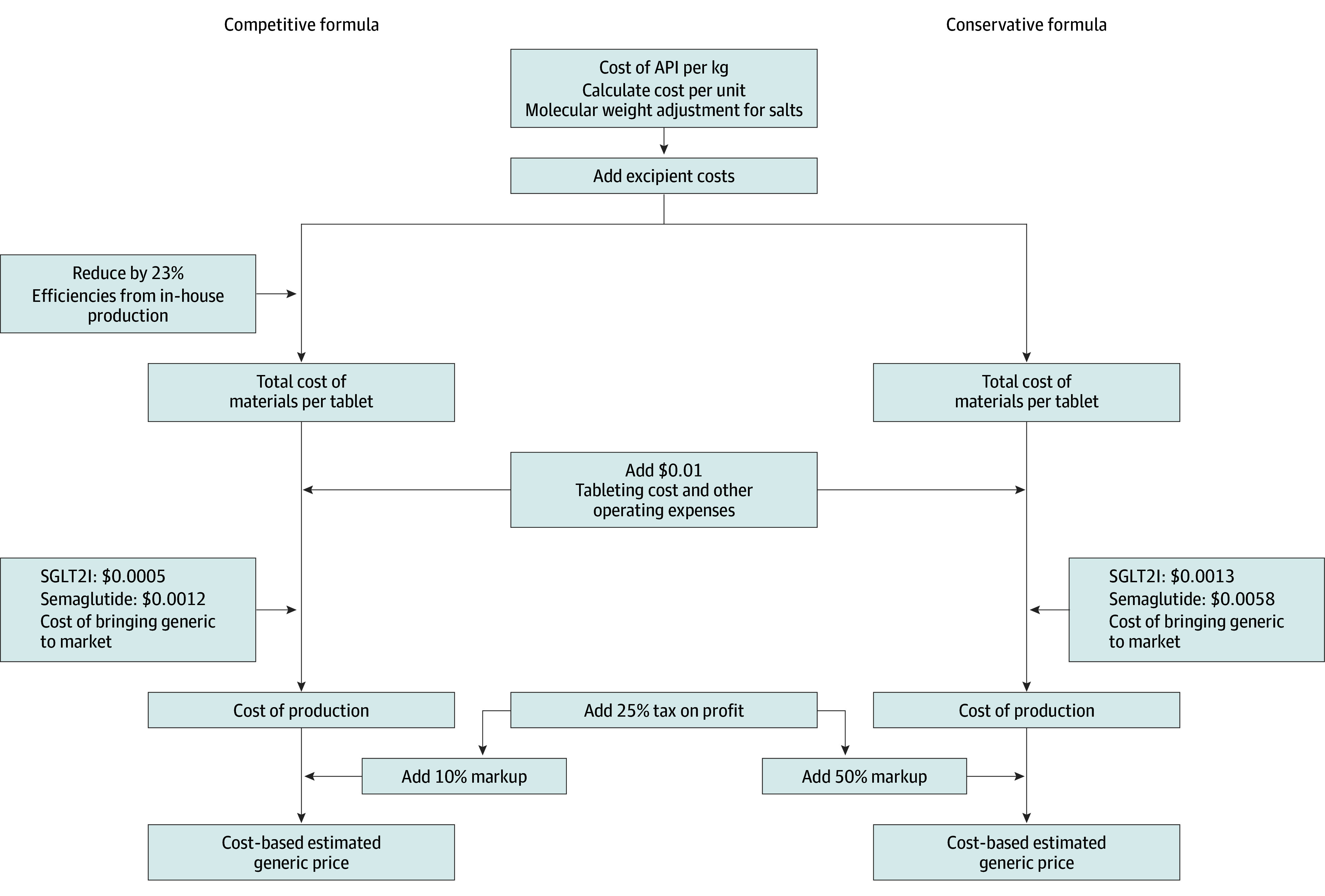
Cost-Based Estimated Generic Price Algorithm for Sodium-Glucose Cotransporter 2 Inhibitors (SGLT2Is) and Oral Semaglutide

The cost of manufacture for medicines in a range of different therapeutic areas has been estimated.^12,13,14^ The methods of these earlier studies served as a starting point for our approach. The cost of the active pharmaceutical ingredient (API) is the first input to which we add the costs of formulation and secondary packaging, logistical costs, profits, and an allowance for tax. A range of CBPs was produced using a competitive formula that assumes large-scale production and a conservative formula that assumes smaller production volumes and/or higher operating or profit margins (Figure 1 and Figure 2). Average API market prices were estimated by statistical analysis of international API shipment data (January 1, 2016, to March 31, 2023) available from a trade database (weighted least-squares regression model) (eMethods in Supplement 1), supplemented with direct solicitation from manufacturers and inference of costs based on product similarity if data could not be identified using the aforementioned means. Costs of specialized injection devices were derived from interviews with industry experts. Further details on API analysis and cost modeling are described in the eMethods and eTables 1-5 in Supplement 1.

Current market prices were collected for 12 countries from public databases (eMethods, eTable 6 in Supplement 1), including 4 high-income countries (France, Latvia, the UK, and the US) and 9 middle-income countries (Bangladesh, Brazil, China [data available only for insulins], El Salvador, India, Morocco, the Philippines, and South Africa). Countries were chosen based on the availability of data on prices and an intention to provide geographic and economic diversity in the sample. We were not aware of a publicly available medicines price database for any low-income country. For each country, we report the lowest price identified for each medicine and each formulation across different manufacturers and package sizes.

### Statistical Analysis

Statistical analyses were performed in R, version 4.2.2 (R Foundation for Statistical Computing). Costs and prices are reported in 2023 US dollars. No inflation adjustment was undertaken as the collected cost inputs represent 2023 costs.

## Results

**Figure 3.**
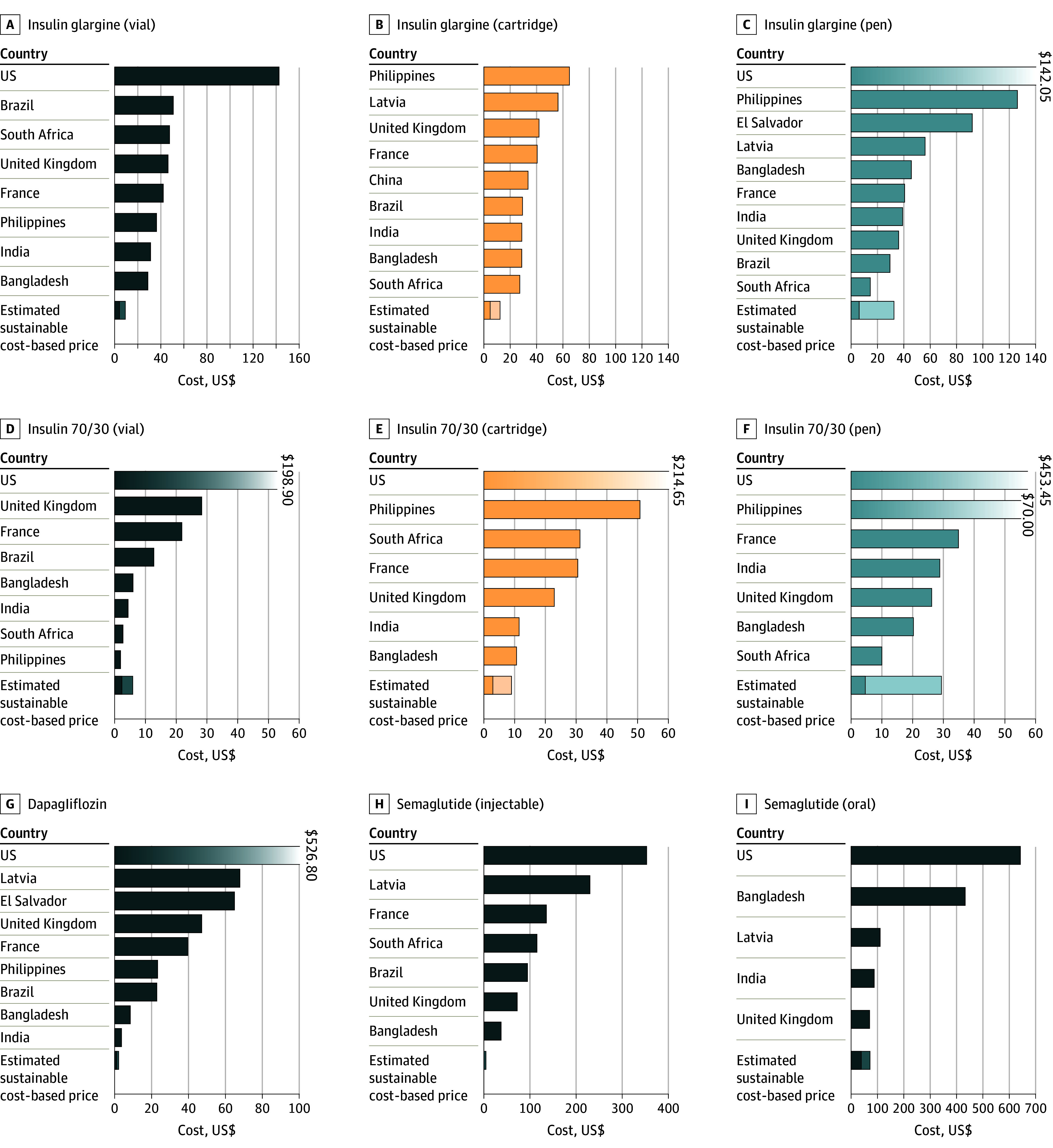
Lowest Market Prices and Cost-Based Prices (per Month, US Dollars) Prices for a medicine per country are not shown if no data were found. The x-axis is scaled to allow best overall visual discernment, and in a small number of cases, prices exceed what can be displayed. Where prices in one country are far higher than most others, that bar is shown with a fading gradient and a data label to indicate the value. The stacked bars for cost-based prices represent the range between the competitive and conservative estimation formulae. Assumptions for calculating per-month prices are the same as outlined in footnote a of Table 1.

When formulated in vials, the CBPs for regular human insulin (RHI) and insulin NPH were between 97% lower to 24% higher than the lowest current market prices, while CBPs for insulin analogues were 25% to 97% lower than the lowest current market prices (Table 1, Figure 3; eFigure 4, eFigure 5, and eResults in Supplement 1). For insulin cartridges, CBPs were 61% to 98% lower than the lowest current market prices, except for detemir, for which the CBP was 38% to 66% lower than the lowest current market prices (Table 1, Figure 3; eFigure 4, eFigure 5, and eResults in Supplement 1). For prefilled pens, CBPs for RHI and insulin NPH were 7% to 88% lower than the lowest current market prices. For insulin analogues, the CBPs for prefilled pens were 52% to 96% lower than the lowest current market prices (Table 1, Figure 3; eFigure 4, eFigure 5, and eResults in Supplement 1). The estimated cost of treatment per person per year was as low as US $61 using twice-daily mixed insulin NPH and US $111 using basal-bolus treatment with insulin glargine and aspart (Table 2).

**Table 1.** Estimated Sustainable Cost-Based Prices, per Month

Medicine^a^	Cost per month, US$, range	
	Cost-based price^b^	Lowest market price
**Insulins**		
Human insulin		
Regular human insulin		
Vial	2.37-5.94	1.93-198.90
Cartridge	3.00-9.13	10.62-53.27
Prefilled	4.69-29.44	9.37-31.73
Insulin NPH		
Vial	2.40-5.98	1.93-198.15
Cartridge	3.02-9.17	10.71-30.70
Prefilled	4.71-29.48	9.37-251.40
Insulin NPH 70/30		
Vial	2.39-5.97	1.93-198.90
Cartridge	3.02-9.15	10.71-214.65
Prefilled	4.71-29.47	9.98-453.45
Insulin analogues (rapid acting)		
Insulin aspart		
Vial	4.86-10.59	19.42-208.35
Cartridge	5.39-13.61	13.95-256.65
Prefilled	7.08-33.92	25.48-268.20
Insulin lispro		
Vial	4.87-10.62	25.18-118.65
Cartridge	5.40-13.63	25.04-488.55
Prefilled	7.09-33.94	26.71-152.70
Insulin glulisine		
Vial	4.79-10.47	21.47-407.70
Cartridge	5.33-13.49	21.24-50.64
Prefilled	7.02-33.81	23.51-526.95
Insulin analogues (long acting)		
Insulin glargine		
Vial	4.25-9.46	28.95-142.65
Cartridge	4.81-12.52	27.47-65.25
Prefilled	6.50-32.83	14.92-142.05
Insulin degludec		
Vial	5.16-11.18	NA
Cartridge	5.69-14.15	51.19-98.55
Prefilled	7.38-34.45	56.33-488.25
Insulin detemir		
Vial	16.97-33.31	443.25-443.25
Cartridge	17.05-35.48	27.47-103.30
Prefilled	18.74-55.79	48.98-443.70
**SGLT2Is**		
Canagliflozin (200 mg daily)	25.00-46.79	17.76-364.60
Dapagliflozin (10 mg daily)	1.30-2.32	3.85-526.80
Empagliflozin (17.5 mg daily)	1.88-3.45	6.08-383.04
**GLP1As**		
Dulaglutide (1.12 mg once weekly)	7.05-17.40	22.20-227.25
Exenatide (7.5 μg twice daily)	0.75-4.46	58.75-577.66
Liraglutide (1.5 mg daily)	21.56-50.32	78.54-851.40
Semaglutide (injectable, 0.77 mg once weekly)	0.89-4.73	38.21-353.74
Semaglutide (oral, 10.5 mg daily)	38.62-72.49	71.15-643.04

Abbreviations: GLP1A, glucagonlike peptide 1 agonist; NA, not available; SGLT2I, sodium-glucose cotransporter 2 inhibitor.

^a^ For insulins, the price when using the typical 100 U/mL formulation is shown, and prices per month in the table assume average use of 50 U/d, based on Médecins Sans Frontières field experience. For SGLT2Is and GLP1 agonists, prices per month shown are based on World Health Organization defined daily doses, which represent a typical dosage (often an assumed average across different dosage regimens), a 30-day month, and use of the most cost-effective dosage form.

^b^ For cost-based prices, the lower end of the range refers to the cost estimated through the conservative formula, and the upper through the competitive formula.

**Table 2.** Costs of Insulin Treatment per Person per Year, With Estimated Sustainable Cost-Based Prices vs Current Market Prices, Including Cost of Injection Devices

Regimen	US$/y, range^a^					
	Syringe and vial		Reusable pens with cartridges		Prefilled pen	
	Cost-based price^b^	Lowest market price	Cost-based price^b^	Lowest market price	Cost-based price^b^	Lowest market price
Basal-bolus regimens						
Insulin NPH BD plus RHI TDS, using 2 reusable pens	157-656	151-2999	96-176	189-575	112-413	169-1777
Insulin glargine OD + insulin aspart TDS	158-589	396-2602	111-213	312-690	127-450	301-2550
Mixed human insulin regimens						
Insulin NPH 70/30 BD	80-306	75-2654	61-138	154-2638	79-380	144-5539
Basal only (T2D)						
Insulin NPH OD	55-189	149-2528	50-127	144-389	68-370	125-1031
Insulin glargine OD	77-232	378-1852	72-168	347-810	90-410	192-1739

Abbreviations: BD, twice daily; OD, once daily; RHI, regular human insulin; T2D, type 2 diabetes; TDS, 3 times daily.

^a^ Prices in the table assume average use of 50 U/d, based on Médecins Sans Frontières field experience. Costs per year include the cost of injection devices (pen or syringe and single-use needles), with assumed costs for these items available in the eMethods in Supplement 1. The cost of each reusable pen is spread over 2 years of use. One reusable pen is used for each type of insulin, so 2 pens are used in regimens in which 2 types of insulin are used.

^b^ For cost-based prices, the lower end of the range refers to the cost estimated through the conservative formula, and the upper through the competitive formula.

Estimated cost-based prices per month were US $1.30 to US $3.45 for SGLT2 inhibitors (except canagliflozin) and US $0.75 to US $72.49 for GLP1 agonists (Table 1). For dapagliflozin and empagliflozin, CBPs were lower than the current lowest market prices, while the CBP of canagliflozin overlapped with the lowest current market prices (Table 1, Figure 3; eFigure 2 in Supplement 1). Cost-based prices per month for GLP1As were all substantially below the lowest current market prices (Table 1, Figure 3; eFigure 3 in Supplement 1). Further details on prices and API cost data used in estimating CBP are presented in the eMethods, eTable 4, eFigure 1, and eResults in Supplement 1.

The greatest international spread of prices was seen for RHI in vials, with a factor of 103 difference across countries (Figure 3). Comparing different formulations for insulins, pen formulations were more expensive per treatment day than vials for 6 of 8 insulins where comparisons were possible (Table 1).

The lowest observed prices for insulin analogues exceeded the CBP by a factor of 1.3 to 38.9 in 12 countries of different income levels (Figure 3). In a minority of cases, the lowest available prices for RHI and insulin NPH were within the range of CBPs (Philippines, South Africa, and India for vials; South Africa for disposable pens).

## Discussion

Our findings suggest that, for nearly all insulins, SGLT2Is, and GLP1As, in nearly all countries surveyed, prices could be reduced substantially if robust generic/biosimilar manufacture was enabled.

### Access to Insulin

It is estimated that only half of the 63 million people with T1D or T2D needing insulin worldwide can access the medicine.^4^ Surveys have reported high rates of insulin rationing even in high-income countries, for reasons including price.^6,7^

Across all insulins, the highest prices were in the US, while the lowest prices were seen in China, France, the Philippines, and South Africa. The lowest observed prices for insulin analogues exceeded the CBP by a factor of 1.3 to 38.9 (Figure 3).

Using a low-cost reusable pen with insulin NPH 70/30 cartridges twice daily could bring annual insulin costs down to $61. Using a basal-bolus regimen of insulin glargine once daily and 3 insulin aspart injections, costs could be as low as $111 (Table 2). These estimates include the costs of insulin, injection devices, and needles, but exclude glucose monitoring, for which reported annual costs range from $98 to $1300.^16^

At present, Médecins Sans Frontières procures insulin for use in humanitarian programs at $3.70 for RHI or insulin NPH or insulin NPH 70/30 in a prefilled pen, $2.48 per RHI cartridge, $2.14 for insulin glargine in a prefilled pen (all containing 300 U), and $2.00 for human insulin in a vial (1000 U). These prices are all within our range of CBP estimates, except for RHI cartridges, for which the CBP was 26% to 76% lower (eTable 7 in Supplement 1).

For all insulins, CBPs were only slightly higher for disposable pens and cartridges compared with vials. However, current market prices were far greater for pen formulations than for vials, suggesting greater markups that are not justified by differences in manufacturing costs.

### Access to SGLT2Is and GLP1As

Current treatment guidelines recommend starting an SGLT2I or GLP1A as soon as T2D is diagnosed in patients with established cardiovascular disease or multiple risk factors for cardiovascular disease or chronic kidney disease.^11^ Based on these guidelines, SGLT2Is and GLP1As would be recommended for a large proportion of patients: for example, one-third of people living with T2D in LMICs have chronic kidney disease,^17^ while 18% in upper middle-income counties and 27% in lower middle-income countries have coronary artery disease.^18^

Compared with insulin, far less literature is available on global access and pricing of SGLT2Is and GLP1As. A 2018 study interviewed experts in Cambodia, India, Pakistan, and Tanzania, finding that access to SGLT2Is and GLP1As was very limited.^8^

Our analysis suggests that major cost reductions could be achieved for the SGLT2Is dapagliflozin and empagliflozin and the GLP1As dulaglutide, exenatide, liraglutide, and oral and injectable semaglutide (Table 1, Figure 3). A recent study of the cost of production for liraglutide and injectable semaglutide as antiobesity treatments, using methods similar to those used herein, produced similar estimates to those in this analysis.^15^

### Limited Competition

Three companies (Novo Nordisk, Eli Lilly, and Sanofi; considered the Big 3) control more than 90% of the global insulin market and 83% of the LMICs market.^19,20^ It has been recognized for years that this oligopoly poses a major barrier to entry for new manufacturers and is a key factor in the lack of access to insulin in many world regions.^21,22^ While at least 40 companies manufacture or market insulin globally, many of these companies operate under licensing or supply agreements with the Big 3. It has been estimated that the number of independent insulin manufacturers is only 10.^21,23^ This limited number of manufacturers has come under government scrutiny: in the US, the state of California has filed a suit against the Big 3 insulin manufacturers, alleging excessive pricing and unfair business practices.^24^

Patents prevent competition and play a leading role in keeping prices high for a wide range of medicines. All SGLT2Is and GLP1As included in this study are under patent protection in the US and Canada, although patents vary in the extent that they block competitors. Generic products are not available in the US, Canada, or the UK, while generic versions are available for all 3 SGLT2Is in India, and a generic/biosimilar version for exenatide is available in India.^25,26^ Biosimilars are currently available in the European Union for insulin glargine, insulin lispro, and insulin aspart.^27^

While most patents covering insulin compounds have expired, secondary patents (ie, patents that cover modifications including formulation, derivates, or method of use) play a role in delaying access to insulin biosimilar products. For example, in the US, plans for launch of one biosimilar insulin glargine product, which had already been approved by the FDA, were aborted following a patent infringement suit.^28^ More than 70 secondary patents have been filed on insulin glargine in the US.^28^ In addition, many insulin injection devices are still covered by patents.^29,30^

### Access to Insulin in Pen Formulations

Approximately 60% of people using insulin globally use (reusable or disposable) pens, with up to 94% using pens in Europe.^31^ The lack of access to devices adds major costs: Médecins Sans Frontières has found that syringes and needles needed to inject insulin cost around $60 per year.^32^

There are, in general, more active patents on insulin devices than on the drug itself.^29^ Our interviews with device manufacturers also reflected a belief that intellectual property on devices was one of the main barriers to market entry for new manufacturers.

In some settings, people living with T1D visit a health facility twice a day to receive insulin. Better access to pen devices could enable increased self-management of T1D in these settings by requiring less training, reducing drug waste, being less prone to dosage errors, and enabling insulin injections outside the home. In 2023, the World Health Organization Essential Medicines List was expanded to include insulin formulation in cartridges and prefilled syringes, due to “ease of use, greater accuracy of dosing, and improved adherence.”^33^

### Analogue vs Human Insulin

With the market share made up by insulin analogues vs human insulin in LMICs steadily increasing,^19^ some have expressed concerns that this will increase costs, as analogues have much higher prices in most cases.^22,34^ This adds urgency to reducing prices for insulin analogues.^35^

Observed API market costs were higher for insulin analogues than for human insulin. However, with manufacturing processes for biologic agents rapidly improving in efficiency and the number of manufacturers increasing, it would not be surprising to see API costs for insulin analogues match or drop below the cost of human insulin API. Already now, in Chinese public tenders, the price of some insulin analogues is lower than prices for RHI or insulin NPH.^36^

### Policy Considerations

Increasing costs for T2D treatment are already placing disproportionate burdens on LMICs: health expenditures for diabetes as a proportion of the gross domestic product are higher in South America, Central America, the Middle East, and North Africa than in Europe.^1^ Additionally, a large proportion of primary care expenditures in LMICs are out-of-pocket, and about half of those expenditures are on medical goods.^37^

In countries with higher prices, major cost savings could be attained through increased availability of lower-cost generics/biosimilars. At the present, this is restrained by a mix of regulatory challenges, intellectual property barriers, and business practices discouraging competition.

Insulins and some GLP1As are biologics. Bringing a biosimilar agent to market is more expensive than for a small molecule (nonbiologic) medicine due to numerous factors, including the requirement to design a new cell line and downstream manufacturing process that yields a similar molecule and, in many cases, requirements to undertake a large clinical trial to prove clinical equivalency.

However, there are reasons to believe that the costs of bringing biosimilars to market may reduce in coming years. The FDA and the World Health Organization have recently updated their guidance for insulin biosimilars, no longer requiring comparative clinical trials if laboratory analyses and pharmacokinetic and pharmacodynamic studies show high similarity.^38,39^

Governments have a range of policy tools, procurement mechanisms, and legal avenues available to reduce the prices of unaffordable medicines. These include, for example, price controls and joint procurement between different countries (pooled procurement). For patented medicines, if agreement on an affordable price cannot be reached in negotiations with a manufacturer, many countries have compulsory licensing provisions in their legislation, which allow for generic importation and manufacture regardless of patents. In some cases, public sector manufacture, as is being pursued in California,^40^ may also be important.

### The Importance of Analyzing Costs of Manufacture

Pharmaceutical prices and manufacturing costs are shrouded in secrecy,^41^ and pharmaceutical manufacturers do not publish breakdowns of manufacturing costs. Analyzing the costs of manufacturing can inform pharmaceutical cost containment policies and procurement negotiations. Some countries have used cost-plus price regulation, applying a formula to calculate a permissible maximum price based on costs of manufacture.^42^

We describe the estimated achievable generic/biosimilar prices presented in this study as sustainable, meaning prices that would be expected in competitive markets that afford manufacturers returns, while avoiding excessive profit margins. Thus, the methods used are not designed to calculate the lowest possible cost of manufacture. Instead, they are based on average costs based on current market rates for key inputs.

Analyzing the cost of production can also help health systems forecast what prices will be possible once generic competition occurs. For example, in the HIV/AIDS pandemic, there were claims that treatment would never be possible in LMICs due to inherently high drug costs.^43^ Once generic manufacturers explained that medicines could be manufactured for under 3% of the original list price, massive treatment programs were established, transforming the deadly pandemic into a manageable chronic disease. A similar pattern was seen for hepatitis C treatments approved over 2014-2017.^43^

A rare glimpse into pharmaceutical companies’ internal cost data is provided in documents submitted by Sanofi to a bipartisan US Senate inquiry, listing the cost of goods sold (COGS) for 5 insulin glargine pens as $7.11 or about $1.42 each (this is listed as mgmt COGS, presumably describing COGS from the management perspective, as opposed to legal COGS).^44^ This is similar to our lower-bound (competitive formula) estimate of $1.20 per pen (Table 2).

### Limitations

This cost-modeling analysis has several limitations. Many components of manufacturing costs are not individually included in cost modeling, including capital investments, quality assurance and control, and regulatory and legal costs. We consider these costs to be accounted for as part of other components (for example, the assumption for cost of biosimilar development and the market costs of API would reflect capital investments and regulatory costs).

The cost of APIs was based on reported values of international shipments. This can be expected to increase the modeled product cost, as it is likely that in-house manufacture or domestic procurement would have lower costs of API.

In nearly all real-world scenarios, additional markups are added to product cost during distribution, such as import tariffs and wholesale distributor markups, which are not specifically included in the model. At the same time, a number of conservative assumptions, especially in the conservative model (giving the top of estimated CBP ranges) will overestimate the price in some settings, as was found for similar models in previous studies.^14^ There is little transparency in medicine prices, and international comparisons continue to be limited and challenging.

## Conclusions

The findings of this economic evaluation suggest that competitive biosimilar manufacture could lower costs: treatment with insulin in a reusable pen device could cost as little as $96 (human insulin) or $111 (insulin analogues) per year for a basal-bolus regimen, $61 per year using twice-daily injections of mixed human insulin (T1D), and $50 (human insulin) or $72 (insulin analogues) for a once-daily basal insulin injection (T2D). With a steadily increasing number of people living with diabetes requiring insulin, strategies must urgently be developed to reduce insulin prices and ensure affordable and reliable access in all parts of the world.

Prices could decrease to $1.30 per month for treatment with SGLT2Is and under $0.75 for treatment with GLP1As. Further price reductions will likely become possible once a robust global generic and biosimilar market emerges.

Given the potential for generic manufacture to substantially reduce prices and thus increase access to these treatments, mechanisms that enabled early generic manufacture in other diseases, such as HIV and hepatitis C, should also be considered for use in diabetes medicines.

## Notes

Supplement 1. **eTable 1.** Summary of Assumed Cost Input Variables **eMethods.** Detailed Methods **eTable 2.** Search Criteria and Salt Forms of Included Medicines **eTable 3.** Criteria Used for Cleaning Export-Import Data **eTable 4.** Costs of Active Pharmaceutical Ingredients **eFigure 1.** Shipment-Level Scatterplots of API Exports **eTable 5.** Bulk Excipient Costs **eTable 6.** National Drug Price Sources **eResults.** Detailed Results **eTable 7.** Sustainable Costs for Diabetes Medicines, by Formulation **eFigure 2.** Lowest Market Prices and Cost-Based Prices (per Month, US$): SGLT2 Inhibitors **eFigure 3.** Lowest Market Prices and Cost-Based Prices (per Month, US$): GLP1 Agonists **eFigure 4.** Lowest Market Prices and Cost-Based Prices (per Month, US$): Human Insulins **eFigure 5.** Lowest Market Prices and Cost-Based Prices (per Month, US$): Insulin Analogues
eReferences

Supplement 2.
Data Sharing Statement
