# Error in Reported Number of Countries

**DOI:** 10.1001/jamanetworkopen.2024.14263

**Published:** 2024-04-22

In the Original Investigation titled “Estimated Sustainable Cost-Based Prices for Diabetes Medicines,”^1^ published March 27, 2024, there was an error in the number of countries reported. Each instance of “13 countries” should have been “12 countries.” In the Methods, the listing of countries from which current market prices were collected given as “Indonesia, Morocco, South Africa, and Ukraine” should have omitted Indonesia and Ukraine and added “the Philippines.” Data from Indonesia were indeterminate and therefore not included in the final analysis. This article was corrected.^1^
