# Supplementary material for: Estimated Sustainable Cost-Based Prices for Diabetes Medicines
Source: JAMA Netw Open. 2024 Mar 27;7(3):e243474. doi: 10.1001/jamanetworkopen.2024.3474 (PMC10973901; doi:10.1001/jamanetworkopen.2024.3474)
Supplement: Supplement 1. — eTable 1. Summary of Assumed Cost Input Variables eMethods. Detailed Methods eTable 2. Search Criteria and Salt Forms of Included Medicines eTable 3. Criteria Used for Cleaning Export-Import Data eTable 4. Costs of Active Pharmaceutical Ingredients eFigure 1. Shipment-Level Scatterplots of API Exports eTable 5. Bulk Excipient Costs eTable 6. National Drug Price Sources eResults. Detailed Results eTable 7. Sustainable Costs for Diabetes Medicines, by Formulation eFigure 2. Lowest Market Prices and Cost-Based Prices (per Month, US$): SGLT2 Inhibitors eFigure 3. Lowest Market Prices and Cost-Based Prices (per Month, US$): GLP1 Agonists eFigure 4. Lowest Market Prices and Cost-Based Prices (per Month, US$): Human Insulins eFigure 5. Lowest Market Prices and Cost-Based Prices (per Month, US$): Insulin Analogues eReferences [file jamanetwopen-e243474-s001.pdf]

Supplementary Online Content

Barber MJ, Gotham D, Bygrave H, Cepuch C. Estimated sustainable cost-based prices for diabetes medicines. *JAMA Netw Open*. 2024;7(3):e243474. doi:10.1001/jamanetworkopen.2024.3474

eTable 1. Summary of Assumed Cost Input Variables

eMethods. Detailed Methods

eTable 2. Search Criteria and Salt Forms of Included Medicines

eTable 3. Criteria Used for Cleaning Export-Import Data

eTable 4. Costs of Active Pharmaceutical Ingredients

eFigure 1. Shipment-Level Scatterplots of API Exports

eTable 5. Bulk Excipient Costs

eTable 6. National Drug Price Sources

eResults. Detailed Results

eTable 7. Sustainable Costs for Diabetes Medicines, by Formulation

eFigure 2. Lowest Market Prices and Cost-Based Prices (per Month, US$): SGLT2 Inhibitors

eFigure 3. Lowest Market Prices and Cost-Based Prices (per Month, US$): GLP1 Agonists

eFigure 4. Lowest Market Prices and Cost-Based Prices (per Month, US$): Human Insulins

eFigure 5. Lowest Market Prices and Cost-Based Prices (per Month, US$): Insulin Analogues

eReferences

This supplementary material has been provided by the authors to give readers additional information about their work.

© 2024 Barber MJ et al. *JAMA Network Open.*

eTable 1. Summary of assumed cost input variables.

**Component**	**Cost per unit (US$)**
API cost	Specific to each medicine, see eTable 4
Vial (10mL)	0.155-0.21
Cartridge (3mL)	0.10-0.33
Disposable pen	0.30-2.50
Reusable pen	5-10
Fill-and-finish and other operating expenses: injectable formulations	0.10
Fill-and-finish and other operating expenses: oral formulations	0.01
Secondary packaging	0.10
Profit	10-50%
Allowance for tax	25%
Needle for pen	0.03
Insulin syringe with needle	0.07-0.32

Sources for these inputs are outlined further below.

© 2024 Barber MJ et al. *JAMA Network Open.*

eMethods. Detailed Methods

Analysis of global API market data

We extracted customs data from a commercial trade database (Panjiva) that systematically archives shipment-level import/export records for numerous countries (eTable 4).
For medicines where no export/import data were available, we estimated API prices through direct solicitation of price quotes from 11 API manufacturers, and inference based on review of methods of synthesis and other information available in academic and trade literature. API manufacturers were identified through internet search, online purchasing platforms (Pharmacompass and Pharmaoffer), supplier listings in cross-country price data collected, and inclusion in an IQVIA report on biosimilar global suppliers.[1] For insulin analogues for which no API data were available, we assumed per-kilogram cost was equal to the API cost for other analogues with available API cost data. Data were collected for shipments from 1 Jan 2019 – 31 Mar 2023. For APIs with fewer than 200 observations (all drugs except insulin RHI and SGLT2 inhibitors), data were collected from 1 Jan 2016 – 31 March 2023.
The Panjiva database reports individual shipments, with a short description of the shipment, date and modality of shipping, shipment size (e.g. in kilograms), value in US$, and origin/destination ports. Data were collected for shipments imported and exported from India, and import data from Bolivia, Brazil, Chile, Colombia, Costa Rica, Ecuador, India, Indonesia, Mexico, Pakistan, Panama, Paraguay, Peru, Philippines, Sri Lanka, Uruguay, USA, and Venezuela. Some key manufacturing hubs (e.g. China) are captured indirectly through this import data. For products where no data were found, a second export-import database was searched.[a]

Data on shipments were manually cleaned to exclude shipments that did not represent genuine API, for example, shipments of finished pharmaceutical product (Appendix Table 2). Shipment of very low weight were also excluded, as these are likely to represent samples or working standards, rather than API bought for formulation. Low weight shipment were defined as those smaller than 10,000 WHO defined daily doses (DDDs). Outliers, defined using the IQR rule, were censored. Weighted least-squares regression was used to fit linear models, with weights corresponding to the volume in kilograms of shipments. The outcome (unit cost) was log-transformed to reduce skew in the residuals. The predicted value on 1 December 2022 was used as the assumed average market price for API. API cost per unit is calculated as milligrams of API per unit multiplied by API cost per kilogram. The amount of API per unit is adjusted for the molecular weight of the hydrate or salt form, where relevant (e.g. canagliflozin hemihydrate).

Statistical analyses were performed in *R* version 4.2.2.

Note on comparison between different types of insulin

[a] This database (Export Genius) has data on some additional countries (Argentina, Bangladesh, Botswana, China, Ethiopia, Ghana, Indonesia, Kazakhstan, Kenya, Lesotho, Liberia, Malawi, Nigeria, Panama, Russia, Tanzania, Turkey, Uganda, Ukraine, USA, Uzbekistan) but does not allow data downloading, and, based on comparison of shipments returned by identical search queries, appears to have a dataset that is less comprehensive than in the Panjiva database.

© 2024 Barber MJ et al. *JAMA Network Open.*

The key manufacturing steps are similar between different insulin analogues and recombinant human insulin.[2–9] While some newer analogues and biosimilars utilize different cell platforms (e.g. *Pichia pastoris*), the use of new cell platforms is driven by a desire to reduce cost of goods sold (COGS), for example, through lower purification requirements.[10,11] For different insulins, it is likely that manufacturing costs are related more to manufacturing capacity (economies of scale) and how modern the equipment used is, rather than differences in the molecular structures. Production capacity (kilograms per year) and yield are the dominant factors in determining COGS for biologic medicines.[12,13] Additionally, biologics manufacturing costs in general have fallen rapidly over the past three decades, as manufacturing equipment and techniques improve.

© 2024 Barber MJ et al. *JAMA Network Open.*

eTable 2. Search criteria and salt forms of included medicines

Drug	Search query	Salt form
**Insulins**		
recombinant human insulin	insuli* AND (huma* OR regul* OR RHI OR IHR	
NPH (neutral protamine Hagedorn) insulin	Insuli* AND ("Neutral protamin* OR Hagedorn OR isophan* OR iso phan* OR NPH)	
mixed insulin 30/70 (30% recombinant human insulin with 70% NPH insulin)		
insulin aspart	insuli* AND asp*	
insulin lispro	insuli* AND lisp*	
insulin glulisine	insuli* AND glul*	
insulin glargine	insuli* AND glar*	
insulin detemir	insuli* AND detem*	
insulin degludec	insuli* AND deglu*	
insulin icodec	insuli* AND icod*	
**SGLT2Is**		
canagliflozin	canaglifloz*	hemihydrate
dapagliflozin	dapaglifloz*	propanediol monohydrate
empagliflozin	empaglifloz*	
**GLP-1 agonists**		
dulaglutide	dulaglutid*	
exenatide	exenatid* OR "exendin 4" OR "synthetic extendin"	
liraglutide	liraglutid*	
lixisenatide	lixisenatid*	
semaglutide	semaglutid*	
semaglutide		
tirzepatide	tirzepatid*	
salcaprozate sodium (SNAC)	salcaprozat* or SNAC	

© 2024 Barber MJ et al. *JAMA Network Open.*

eTable 3. Criteria used for cleaning export-import data

Reason for censoring	Example of product description that would trigger censoring
Incorrect substance identified	"insulin aspart" (in a search for insulin detemir)
Quantity of shipment	Value listed in "quantity" variable less than 100,000 DDD in kg
Finished Pharmaceutical Product (FPP)	"insulin degludec tresiba penfill 100U/mL"
Impurity	"insulin glargine (impurity precursor)"
Unclear quantity of API	"dapagliflozin granules"
Free sample or 'no commercial value'	"insulin human isophane NPH no commercial value"
Reference/working standard	"canagliflozin working standard"

Note: Export data were downloaded from a commercial trade database (Panjiva). See note Analysis of global API market data for more information on the sources and structure of this data source.

© 2024 Barber MJ et al. *JAMA Network Open.*

eTable 4 summarizes data on API shipments and modelled average API prices, and bubble plots visualising API shipment data are shown in eFigure 1.

Among insulins, global API data were available for RHI, NPH, and glargine. Limited data (n=4) were available for aspart. Results from weighted least-squares regression suggest predicted average prices on 1 December 2022 of US$31,169/kg for recombinant human insulin (RHI), 29,732 US$/kg for NPH, 65,665 US$/kg for glargine, and 81,164 US$/kg for aspart. No data were observed for insulin degludec, detemir, or glulisine. We received price quotes from 1 manufacturer for dulaglutide and liraglutide.

There were data on 671 shipments of SGLT2Is. Average API prices were 2,283 US$/kg for canagliflozin, 1,468 US$/kg for dapagliflozin, and 1,787 US$/kg for empagliflozin.

Per kilogram costs for GLP1A API were 542,500 US$/kg for exenatide, 573,521 US$/kg for liraglutide, and 70,569 US$/kg for semaglutide. We received quotes from manufacturers for liraglutide (500,000 US$/kg) and dulaglutide (1,500,000 US$/kg).

No data on API shipments were found for insulin icodec, lixisenatide, or tirzepatide.

Where both price quotes received from manufacturers and exim data were available, we assumed the lower price for the purposes of calculating a sustainable cost-based price.

Note on exenatide

For exenatide, all observed API was shipped by the same company, with prices ranging 542,500 US$/kg to 1,007,140 US$/kg, with more costly API destined for high income countries in Western Europe and lower-priced API observed sold to less wealthy countries. The most recent shipment was twice as expensive as shipments observed to less wealthy destinations, resulting in the linear prediction model showing a sharp increase in price. Given that the product description and company are identical, we assume that this is a tiered price model, and use the lower level (542,500 US$/kg) as the API price.

© 2024 Barber MJ et al. *JAMA Network Open.*

eTable 4. Costs of active pharmaceutical ingredients

Medicine	Observations	Cost of API (US$/kg)	Source
Insulins			
RHI	317	31,169	Export-import data
NPH insulin	0	24,986	Export-import data; combination of RHI API cost and protamine cost
insulin aspart	4	81,164	Export-import data
insulin lispro	0	81,164	Assumed based on costliest observed analogue (aspart)
insulin glulisine	0	81,164	Assumed based on costliest observed analogue (aspart)
insulin glargine	289	65,665	Export-import data
insulin detemir	0	81,164	Assumed based on costliest observed analogue (aspart)
insulin degludec	0	81,164	Assumed based on costliest observed analogue (aspart)
**SGLT2Is**			
canagliflozin	24	2,283	Export-import data
dapagliflozin	277	1,468	Export-import data
empagliflozin	370	1,787	Export-import data
**GLP1As**			
dulaglutide	0	1,500,000	Manufacturer quote
exenatide	8	542,500	Export-import data
liraglutide	9	573,521	Export-import data
		500,000	Manufacturer quote
lixisenatide	0	No data	
semaglutide	21	70,569	Export-import data
tirzepatide	0	No data	

© 2024 Barber MJ et al. *JAMA Network Open.*

eFigure 1. Shipment-level scatterplots of API exports.

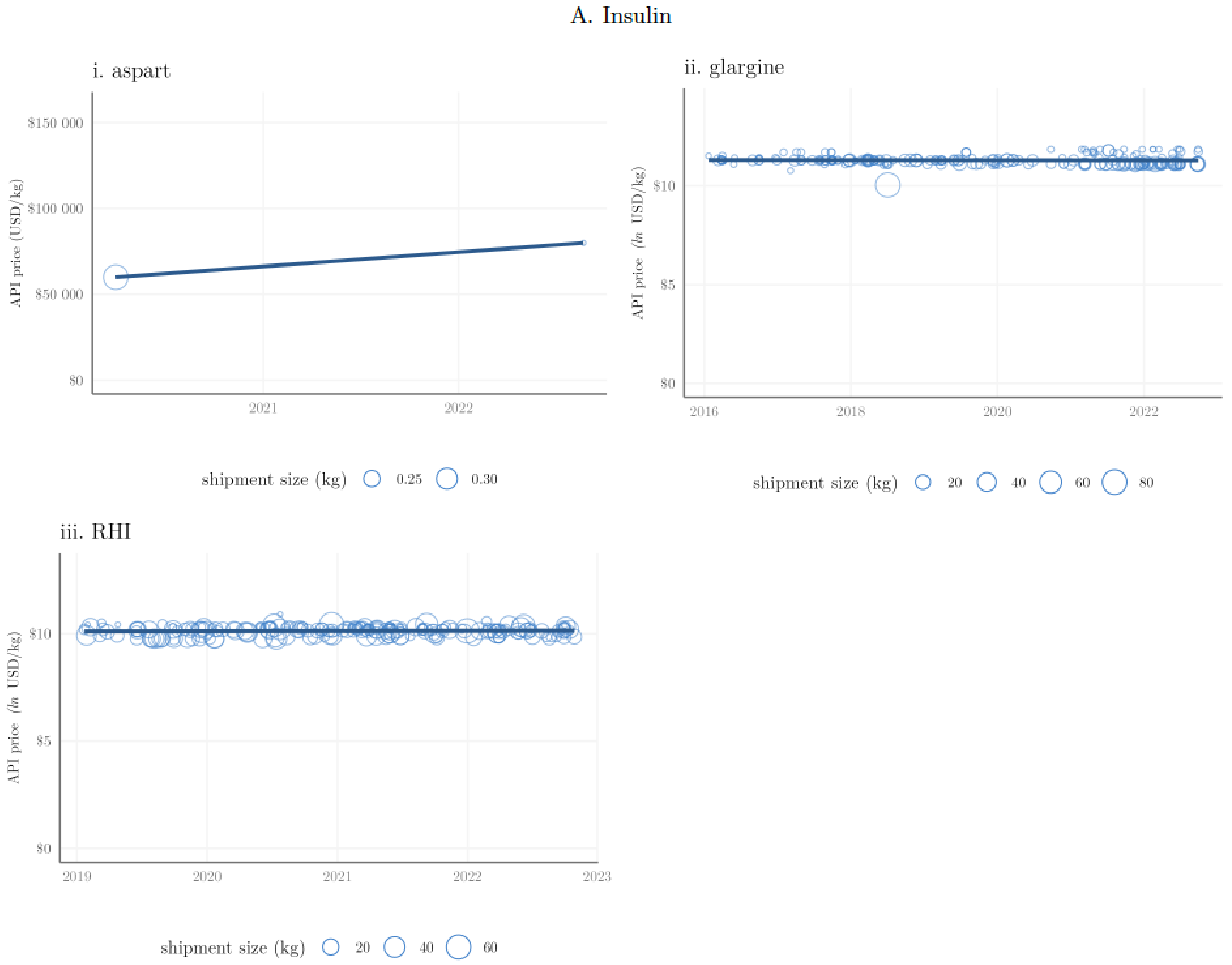

© 2024 Barber MJ et al. *JAMA Network Open.*

B. GLP-1 agonists

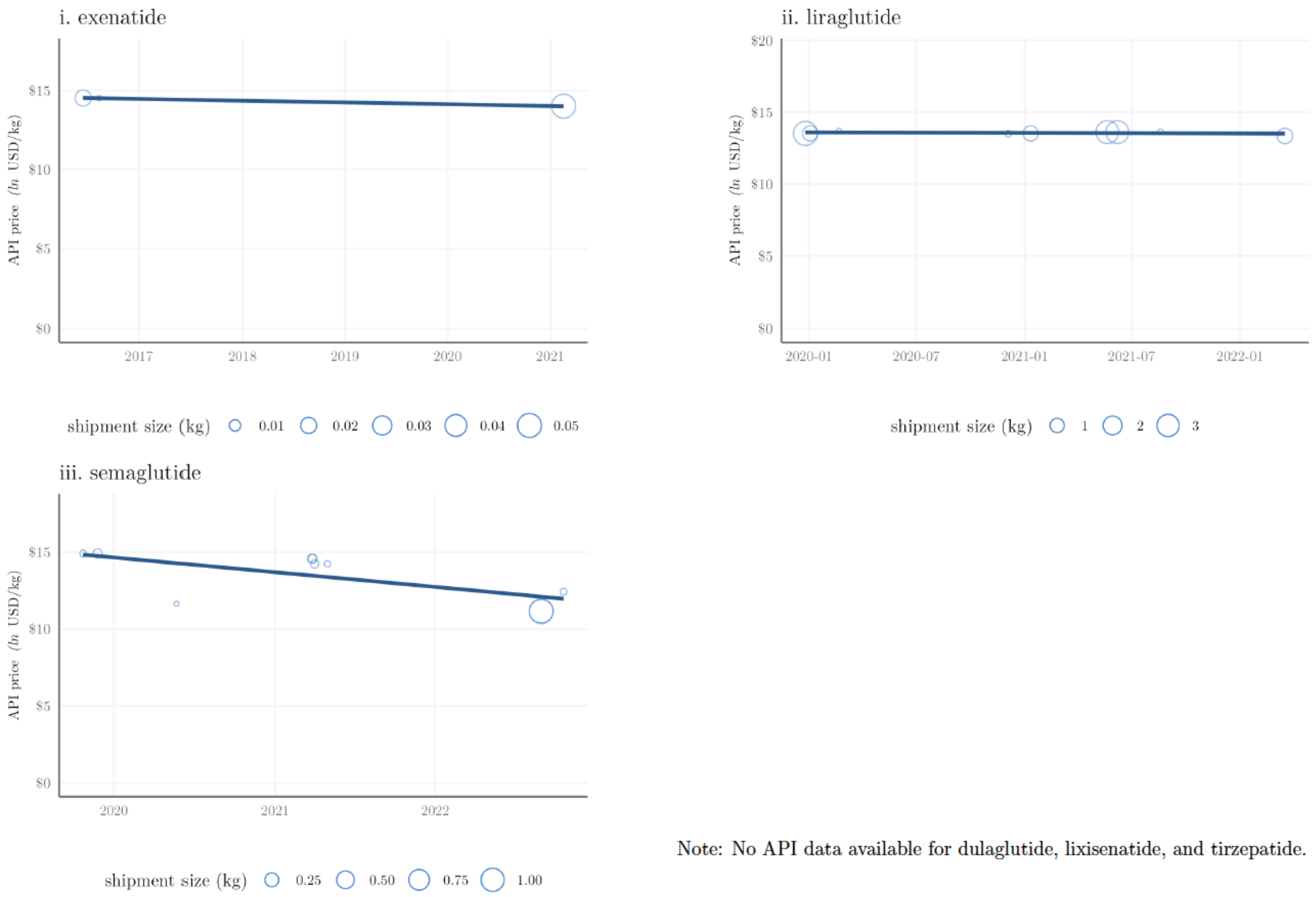

Note: No API data available for dulaglutide, lixisenatide, and tirzepatide.

© 2024 Barber MJ et al. *JAMA Network Open.*

C. SGLT-2 inhibitors

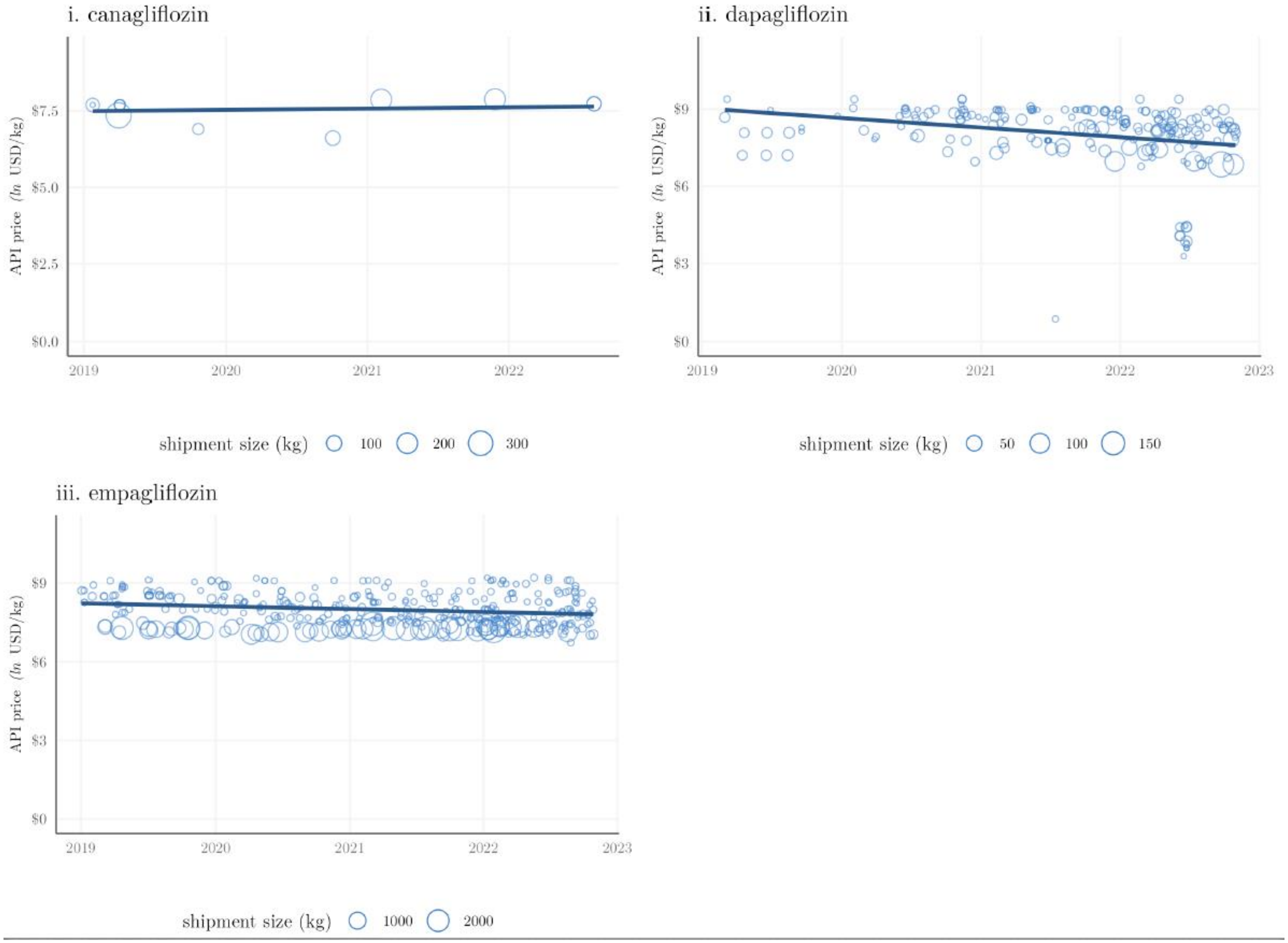

© 2024 Barber MJ et al. *JAMA Network Open.*

Analysis of in-house versus third-party API costs

API can be manufactured in-house or purchased by a third party and processed in final pharmaceutical product (FPP) facilities. API observed on global markets includes both, with third-party API inherently including a profit margin. In the competitive formula, we assume lower costs of API possible with in-house production.

The shipments of API that form the basis for our analysis can be divided into two types: a) sales from an API manufacturer to a separate, overseas pharmaceutical company that formulates the API into a finished pharmaceutical product (FPP), effectively 'outsourced API', or b) API that is manufactured by one subsidiary and shipped to another subsidiary in the same multinational pharmaceutical corporation, termed 'in-house' manufacture. A sub-analysis was performed for those API shipments that indicated API is 'in house'. To estimate this parameter, we used a general linear model to estimate the average cost of in-house API relative to third-party market API (identified through export records). The model is outlined further below.

In-house shipments were identified through export declarations, which sometimes include 'in-house' with the description. Some in-house descriptions may be undeclared, but assuming that likelihood of declaring 'in-house' versus not declaring for in-house treatment is not correlated to price, then any bias would result in a conservative (i.e. underestimate) of the price discount associated with in-house production. 'In-house' is a relatively rare declaration in exim data; it only occurs when a firm separates API and fill-and-finish production in different countries which have exim data availability. To increase the sample, we used a convenience sample of all API exim data cleaned in the last year across other projects. This included 60,874 observations across 43 drugs, representing a wide range of therapeutic categories, formulation types, and current market costs. These drugs included: acetaminophen, amlodipine, artenunate, azithromycin, baricitinib, bedaquiline, bisoprolol, budesonide, dexamethasone, doxycycline, gliclazide, hydrochlorothiaide, hydrocortisone, hydroxychloroquine, imatinib, ivermectin, linezolid, lisinopril, lopinavir, losartan, metformin, methylprednisolone, moxifloxacin, prednisolone, remdesivir, rifapentine, ritonavir, ruxolitinib, simvastatin, tofacitinib, and all diabetes medicines examined in this paper.

$$lnY_{it} = \alpha_i + \beta_1 time + \beta_2 volume + \beta_3 inhouse + \epsilon_{it}$$

The model accounts for time trends and fixed-level effects for individual APIs. In this model, lnY_{it} is the log-transformed price of API *i* at time *t*, β_1 is the time trend, β_2 adjusts for confounding by volume of shipment size, and in-house is an indicator variable that is 1 when the shipment is declared as in-house and 0 when it is not. Standard errors are clustered at the API level.

© 2024 Barber MJ et al. *JAMA Network Open.*

Regression summary:

Observations: 60,874
Fixed-effects: API: 66

term	estimate	standard error	*t* value	p value
time	0.00	0.00	0.11	0.9151
volume	-0.10	0.01	-9.72	0.0000***
1 if in-house, 0 if not	-0.23	0.08	-2.73	0.0081**

*(p < 0:001***) ; (p < 0:005**) ; (p < 0:05*)*

RMSE: 0.55476 Adj. R2: 0.936251 Within R2: 0.064619

This analysis found that API manufactured in-house was priced 23% lower than outsourced API. This cost saving was assumed in the “competitive” costing algorithm (Figure 1 in main text).

© 2024 Barber MJ et al. *JAMA Network Open.*

Capital and operating expenditures, logistics, taxes, and mark-up

Manufacturing any product requires capital expenditures, such as up-front investments to build a manufacturing plant. Some earlier analyses of health product manufacturing costs account for capital expenditure separately from ongoing costs (operating expenditure).[14] The cost inputs used for API, vial, cartridge, and pen device costs represent commercial prices on current markets, obtained through review of commercial data and informant interviews (eTable 1). These prices (assuming the manufacturers are not selling at a loss) cover the relevant capital expenditures, and, as we argue below, likely cover most operating expenditures.

The cost of fill and finish for parenteral formulations was based on currently available prices of simple injectable products – water for injection (WFI) and sodium chloride. The lowest cost vials of WFI or sodium chloride are priced around US$0.10 per unit. The price of these products includes raw materials costs, costs of sterile formulation, costs of secondary packaging, quality standards of a stringent regulatory authority (SRA), and a profit margin. As raw material costs and profit margins are likely to be very low for these products, we conservatively assume that this price represents the cost of sterile formulation alone, and add this to the costs of primary packaging materials. For an upper-bound assumption representing lower volume manufacture, we double this cost to US$0.20 per unit. We assumed 7% overfill for vials, following USP recommendations.[15] We assumed 3% overfill for prefilled syringes.[16] We assumed 3% overfill for cartridges, by analogy to prefilled syringes, which require little to no overfill.[16]

For solid oral formulations (tablets and capsules), we assumed a formulation cost of US$0.01 per unit, based on our previous work, which reflects capital and operating expenditures for a plant formulating API into solid oral formulations.[17]

A range of 10-50% was assumed for profit margin. The corporate tax rate was assumed to be 25%, the global average.[18]

© 2024 Barber MJ et al. *JAMA Network Open.*

Cost of vials, cartridges, and pen devices

Insulin can be injected in three ways: using a vial and insulin syringe, disposable pen (a pen pre-filled with an insulin-containing cartridge, with the entire device discarded once the cartridge is empty), or a reusable pen that accepts cartridges. In all cases, a new needle must be used for each injection. GLP1As are injected using disposable pens pre-filled with cartridges, except for the oral formulation of semaglutide.

We contacted manufacturers of injection devices, identified through review of trade magazines. There are relatively few actors in this sector, and our interviewee sample represents a substantial proportion of active firms. Interviewees reported that there were no more than 14 total manufacturers active in these markets; this estimate is in line with trade reports that identify 10-17 major global insulin pen manufacturers.[19,20] We identified 22 potential interviewees on the basis of work experience, identified through trade magazines, LinkedIn, or snowball referrals. Six consented to be interviewed for this publication, having experience in 11 companies, including former employees or consultants from all 'Big 3' insulin companies. Representatives of Sanofi did not respond to emails requesting interviews. Eli Lilly and Novo Nordisk declined to be interviewed, with an employee of Lilly writing that they do not "typically engage in external discussions around the costs of our insulin or injection [devices]" and an employee of Novo Nordisk writing that "in general Novo Nordisk is not sharing any input on [their] manufacturing cost". Interviewees were informed that any information shared would be reported in a way that would not be identifiable to them. In some cases, we followed up with interviewees if clarifications were needed, and to ensure that they were comfortable with the degree of detail published in this study. In some cases, manufacturers were willing to give specific quotes for prices at given volumes.

Vial material costs

We assumed that the material costs for glass vials range US$0.155-0.21, based on Clendinen 2016 and CEPI 2020.[14,21]

Disposable pens

One interviewee estimated that the cost of manufacturing disposable pens at large volumes is in the range US$0.30-0.70 per unit, while cost at lower volume would be US$1.00-2.00 per unit.

One interviewee estimated that the cost of manufacturing disposable pens at large volumes is US$0.50-0.70 per unit.

One interviewee reported that the price offered by a large European-based devices company would be US$1.2-2.5 for an order quantity of 0.5-1 million units.

Reusable pens

One interviewee mentioned US$20 per reusable pen (ex works) at a volume of 1 million per year.

© 2024 Barber MJ et al. *JAMA Network Open.*

One interviewee mentioned that COGS for high-quality reusable pens manufactured in Germany is around US$15 per unit. One interviewee mentioned that a reusable plastic pen that is made in India for the Indian market would have COGS below US$5 per unit.

One interviewee reported that the price offered by a large European-based devices company would be US$8-10 for an order quantity of 0.5-1 million units.

One interviewee, who had undertaken a market survey for prices of reusable pens available for purchase online, reported a range of US$8-58 per unit.

While we did not undertake an extensive search for current prices for reusable pen devices, two examples are the Chinese manufacturer Delfu citing a price of US$8-12 per unit for ≥1000 unit order quantity (see https://www.delfu-medical.com/sale-2800707-manual-plastic-diabetes-insulin-pen-for-prefilled-3ml-cartridge.html), and the GensuPen 2, manufactured in Poland, sold for around US$6 per unit online (see https://allegro.pl/listing?string=gensupen).

Cartridges

One interviewee reported that, for a large European-based devices company, the cost of a cartridge (without API) would range between US$0.10-0.20.

Delfu, a large manufacturer in China, advertises a bulk price of US$0.13-0.33 (https://www.delfu-medical.com/sale-2800725-insulin-pen-cartridges-and-vials-for-pharmaceutical-packaging.html)

Secondary packaging

We assumed that secondary packaging costs US$0.10 per unit for injectable formulations, based on Clendinen 2016.[14]

For solid oral formulations, the cost of packaging is captured in the 'conversion cost' of US$0.01 per tablet/capsule, following Hill et al 2018.[17]

Needles for pen devices

Disposable needles for insulin pens are available for bulk purchase at US$0.03 per unit and less (https://www.delfu-medical.com/sale-2825122-disposable-safety-insulin-pen-needles.html , https://www.alibaba.com/trade/search?IndexArea=product_en&CatId=&fsb=y&viewtype=&tab=all&SearchScene=&SearchText=30g+needle+insulin)

Insulin syringes with needles

This cost component is used only for calculating the cost per patient per year for insulin delivered by vial and syringe versus by a pen device, in Table 2 of the main text. MSF reported that their projects procure insulin syringes with a luer-lock needle at US$0.07 per unit (

© 2024 Barber MJ et al. *JAMA Network Open.*

https://msfaccess.org/sites/default/files/2022-05/Diabetes_NCDs_TechBrief_AC-SD_Diabetes-resolution_ENG_May2022.pdf), which we use as the lower-bound assumption. To provide an upper-bound assumption, we use the mean cost of procurement reported in a survey of 37 centers providing diabetes care in LMICs, of US$0.32 (Klatman EL, Ogle GD. Access to insulin delivery devices and glycated haemoglobin in lower-income countries. *WJD* 2020; **11**: 358–69.).

GLP1 injection pens

GLP1 injection pens are analogous to disposable pens for insulins, containing the drug in a pre-loaded cartridge (see for example page 200, https://www.accessdata.fda.gov/drugsatfda_docs/nda/2017/209637Orig1s000OtherR.pdf), and we make identical cost assumptions.

© 2024 Barber MJ et al. *JAMA Network Open.*

Estimating costs per person per year

The combined yearly cost of insulin treatment can be calculated. While insulin requirements vary, WHO suggests a standard daily dose of 40 units is used for comparisons.[22] This would equal 1,200 units per 30 days. However, calculation on this basis represents 'perfect' use (for example, with no wasted insulin). MSF field analyses suggest real-world usage is closer to 1,500 units per month among people living with T1D and T2D receiving care in MSF projects;[23] we have therefore assumed 1,500 units per month for both T1D and T2D for the purposes of this high-level comparison, equivalent to 60 300-unit cartridges per year. For basal-bolus regimens, we assumed that insulin dosage is split equally between basal and rapid-acting insulins, i.e. 25 units of each per day.

The cost of a reusable pen is added by spreading a cost of US$5-10 (eTable 1) over two years, or 0.7-1.4 cents a day. The cost of needles at US$0.03 per unit (eTable 1) is added as two needles per day when using mixed insulin, and four needles per day when using a basal-bolus regimen (1 basal and 3 bolus injections). The cost of an insulin and syringe (when drawing insulin from a vial) is added as US$0.07-0.32 per injection, at the same daily use as above. For most people using insulin for T2D, a single daily injection of long-acting insulin will be used.

Cost of developing biosimilars and generics

For insulins and GLP1As, we added costs for biosimilar development. Developing a biosimilar is more expensive than developing a generic version of a small-molecule medicine (see also in *Discussion*). In our earlier analysis, we used an assumption of US$100 million per biosimilar, which we noted was likely conservative (high).[24] Since then, new data have been published, including actual development costs reported by biosimilar insulin manufacturers. Analysis by the Medicines Patent Pool (MPP) estimated that the cost of bringing biosimilars to market ranges US$4-53 million, with the top of the range representing monoclonal antibodies, which are significantly more complicated than smaller biologics like insulin or GLP1As.[25] Additionally, a Chinese biosimilar insulin manufacturer has published breakdowns of development costs, indicating a total cost of US$11 million for development of an insulin glargine biosimilar,[26] which falls within the range estimated by MPP.[26] We assumed that biosimilar development costs range US$11-53 million, and are recouped over five years. Assuming 1 million consumers for a new biosimilar insulin (about 3% of estimated insulin users globally),[27] this adds a cost of US$2.2-10.6 per consumer per year, over five years.

For GLP1As, we assumed a biosimilar market of 5 million consumers for a new biosimilar GLP1A. In addition to being indicated for a significant proportion of people with T2D, GLP1As are, GLP1As are generally indicated for people with BMI >30kg/m^2, or around 650 million adults worldwide.[28] The assumed 5 million consumers would thus represent just under 1% of clinically eligible adults globally.

For generics of SGLT2Is, which are small molecules, we assumed development costs based on a 2021 analysis reporting a modelled average cost of US$2.4 million per generic, of which US$1 million is attributed to API development, and around US$400,000 is budgeted for FDA

regulatory submissions.[29] We use US$2.4 million as the upper-bound (conservative) estimate, and assume a lower-bound (competitive) estimate of US$1 million, based on much generic manufacture taking place in LMICs, where costs are lower, and considering that in the scenario modelled here, already-developed generic API would be used.

As for insulin, we assumed 1 million consumers for a new generic SGLT2 inhibitor. Given the number of patients clinically eligible for SGLT2 inhibitors is likely to be substantially greater than for insulin, this is even more conservative as an assumption.

Costs of excipients

Excipients are ingredients other than the API which are added in a given formulation. Excipient prices were sourced from bulk commercial suppliers. Excipient information was sourced from package inserts, published information related to regulatory approval, or trade literature. Commercial suppliers are generally for lab use rather than high volume manufacturing. These costs are therefore conservative.

eTable 5. Bulk excipient costs.

Item	Price (US$/kg)	Reference
Disodium hydrogen phosphate dihydrate	35.7	https://www.sigmaaldrich.com/US/en/substance/disodiumhydrogenphosphatedihydrate1779910028247
Glycerin	69.2	http://www.sigmaaldrich.com/catalog/product/sial/g2289?lang=en&region=US
glycerol	65.5	https://www.sigmaaldrich.com/US/en/substance/glycerolsolution9209anhydrousbasis56815
M-Cresol	40.0	http://www.emdmillipore.com/US/en/product/m-Cresol,MDA_CHEM-809691
MEG-1	90.9	https://www.sigmaaldrich.com/SE/en/product/sial/324558
Phenol	192.0	http://www.emdmillipore.com/US/en/product/Phenol,MDA_CHEM-822296
Polysorbate-20	613.0	http://www.sigmaaldrich.com/catalog/product/sial/44112?lang=en&region=US
Polysorbate-20	613.0	https://www.sigmaaldrich.com/US/en/substance/polysorbate20123459005645
Protamine sulfate	15200.0	https://www.sigmaaldrich.com/US/en/product/sigma/p3369
Sodium choloride	8.8	https://www.sigmaaldrich.com/US/en/product/mm/106400
Sodium phosphate dibasic	31.0	https://www.sigmaaldrich.com/US/en/substance/sodiumphosphatedibasic141967558794
Sodium phosphate dibasic dihydrate	92.4	https://www.sigmaaldrich.com/US/en/substance/sodiumphosphatedibasicdihydrate1779910028247
Tromethamine	146.4	https://www.sigmaaldrich.com/US/en/substance/tromethamine1211477861
Water for injection	11.1	http://www.sigmaaldrich.com/catalog/product/mm/486505?lang=en&region=US

© 2024 Barber MJ et al. *JAMA Network Open.*

Zinc oxide	170.0	http://www.sigmaaldrich.com/catalog/product/sigald/14439?lang=en&region=US

All hyperlinks accessed January 14, 2023.
HCl, NaOH, NaOAc, glacial acetic, and mannitol acid used for buffering or tonicity-adjusting not included in excipient cost calculation.

Excipient content per mL

Except where indicated otherwise, composition of vials is from Niazi SK. Handbook of Pharmaceutical Manufacturing Formulations, Third Edition: Sterile Products. Boca Raton, FL: CRC Press, Taylor & Francis Group; 2020.

Insulin aspart

Component	Cost per kg (US$)	Amount per 1mL	Cost per 10mL vial (US$)
Glycerin	69.2	16mg	0.01107200
Phenol	192	1.5mg	0.00288000
M-Cresol	40	1.72mg	0.00068800
Zinc oxide	170	19.6mg	0.03332000
Disodium hydrogen phosphate dihydrate	35.68	1.25mg	0.00044600
Sodium chloride	8.82	0.58mg	0.00004410
Water for injection	11.1	1g	0.11100000
		Total:	0.15945010

Insulin degludec
From:
https://www.accessdata.fda.gov/drugsatfda_docs/label/2018/203314s008lbl.pdf

Component	Cost per kg (US$)	Amount per 1mL	Cost per 10mL vial (US$)
Glycerol	65.5	19.6mg	0.01284303
Phenol	192	1.5mg	0.00288000
metacresol	40	1.72mg	0.00068800
zinc	170	71.9mg	0.12223000
Water for injections	11.1	1g	0.11100000
		Total:	0.24964103

Insulin detemir
From Niazi SK. Biosimilars and Interchangeable Biologics: Strategic Elements. CRC Press. 2016.

Component	Cost per kg (US$)	Amount per 1mL	Cost per 10mL vial (US$)
meg inc	90.9	65.4 mg	0.05944564
m-cresol	40	2.06mg	0.00082400
glycerol	65.5	16mg	0.01048411
phenol	192	1.8mg	0.00345600
disodium phosphate dihydrate	92.4	0.89mg	0.00082236

© 2024 Barber MJ et al. *JAMA Network Open.*

sodium chloride	8.82	1.17mg	0.00010319
Water for injection	11.1	1g	0.11100000
		Total:	0.18613530

Insulin glargine

Component	Cost per kg (US$)	Amount per 1mL	Cost per 10mL vial (US$)
Zinc oxide	170	30mg	0.05100000
M-Cresol	40	2.7mg	0.00108000
Glycerol 85%	65.5	20mg	0.01310513
Water for injection	11.1	1g	0.11100000
		Total:	0.17618513

Insulin glulisine

From Niazi SK. Biosimilars and Interchangeable Biologics: Strategic Elements. CRC Press. 2016.

Component	Cost per kg (US$)	Amount per 1mL	Cost per 10mL vial (US$)
Metacresol	40	3.15 mg	0.00086000
Tromethamine	146.4	6 mg	0.00878400
Sodium chloride	8.82	5mg	0.00044100
Polysorbate-20	613	0.01mg	0.00006130
Water for injection	11.1	1g	0.11100000
		Total:	0.12114630

Insulin lispro

Component	Cost per kg (US$)	Amount per 1mL	Cost per 10mL vial (US$)
Protamine sulfate	15,200.00	0.28 mg	0.04256000
Glycerin	69.2	16.00 mg	0.01107200
Sodium phosphate dibasic	31	3.78mg	0.00117180
M-Cresol	40	1.76mg	0.00070400
Zinc ion	170	0.025mg	0.00004250
Liquefied phenol	192	0.715mg	0.00137280
Water for injection	11.1	1.00g	0.11100000
		Total:	0.16792310

Insulin NPH

Component	Cost per kg (US$)	Amount per 1mL	Cost per 10mL vial (US$)
Zinc oxide	170	0.012mg	0.00002040
Liquefied phenol	192	0.73mg	0.00140160
Metacresol	40	1.6mg	0.00064000
Glycerin	69.2	16mg	0.01107200
Protamine sulfate	15200	0.35mg	0.05320000
Sodium phosphate dibasic	31	3.78mg	0.00117180
		Total:	0.06750580

© 2024 Barber MJ et al. *JAMA Network Open.*

Regular human insulin

Component	Cost per kg (US$)	Amount per 1mL	Cost per 10mL vial (US$)
Metacresol	40	2.5 mg	0.00100000
Glycerin	69.2	16 mg	0.01107200
Water for injection	11.1	1g	0.11100000
		Total:	0.12307200

© 2024 Barber MJ et al. *JAMA Network Open.*

Special formulations (insulin NPH, oral semaglutide, once-weekly exenatide)

Insulin NPH is produced by mixing human insulin with protamine, zinc, and phenol. Market costs for protamine, zinc, and phenol were added to the cost of human insulin API to estimate insulin NPH API costs.

Semaglutide is a biologic, first approved in injectable form. In 2019, a new oral formulation of semaglutide was approved, making it the first approved oral GLP1 agonist,[30] and one of few available biologics that are orally bioavailable. The oral bioavailability of the product is made possible primarily by using the excipient salcaprozate sodium (or SNAC), which protects semaglutide from degradation in gastric acid.[30] Each tablet of oral semaglutide contains 300mg SNAC, independent of semaglutide dosage; available formulations are semaglutide/SNAC 3/300mg 7/300mg 14/300mg.[31] We extracted data on SNAC market prices as for API, described above.

Twice-daily and once-weekly formulations are available for exenatide. The long-acting pharmacokinetics of once-weekly exenatide are enabled by encapsulating exenatide in PGLA microspheres.[32] We did not include the long-acting formulation of exenatide in this analysis, due to challenges in accounting for the cost of the PGLA formulation process.

Dosage of insulins in milligrams

Regular human insulin 100 units = 3.50mg
Insulin NPH 100 units = 3.91mg
Lispro 100 units = 3.50mg
Aspart 100 units = 3.50mg
Glulisine 100 units = 3.49mg
Glargine 100 units = 3.64mg
Detemir 100 units = 14.20mg
Degludec 100 units = 3.66mg
Source: Summary of Product Characteristics available from www.medicines.org.uk.

Insulin NPH contains 0.35mg protamine per 100u human insulin.[33]

Current market prices

Current market prices were collected from publicly available sources for 13 countries (eTable 6) in January 2023. For each product and formulation in each country, the lowest available price was recorded. Vial-sharing and pill-splitting were permitted, with WHO DDDs are used for standardization for SGLT2Is and GLP1As, and MSF field usage data (1500 units per month) used for insulins. Where multiple databases are available for one country (e.g. for government procurement versus private market), we used the lowest price available across databases. Where different types of prices are reported (e.g. price for sale to the government versus price for sale to pharmacies, price including or excluding tax), we used the lowest available price.

© 2024 Barber MJ et al. *JAMA Network Open.*

eTable 6. National drug price sources.

Country	Income category*	Source	URL	Notes
Bangladesh	Lower-middle	Medex	https://medex.com.bd/	This database lists retail prices.
Brazil	Upper-middle	Agência Nacional de Vigilância Sanitária	https://www.gov.br/anvisa/pt-br/assuntos/medicamentos/cmed/precos	Preço Fabrica (maximum price for sale to pharmacies) used at 0% tax rate. Only products that are listed as commercialised in 2022 were included.
China	Upper-middle	National insulin tender	https://www.smpaa.cn/gjsdcg/files/file7793.pdf	This data only includes insulin.
El Salvador	Lower-middle	Gobierno de El Salvador. Listado de Precios de Venta Máximo al Publico de Medicamentos	http://info.medicamentos.gob.sv/	Maximum Sale Price to the Public (PVMP) used.
France	High	Base de données publique des médicaments	https://base-donnees-publique.medicaments.gouv.fr/index.php	Only nationally reimbursed products included.
India	Lower-middle	1mg.com	https://www.1mg.com/	This database lists retail prices. Assumed that discount for purchasing online is applied.
Latvia	High	State Agency of Medicines	https://www.zva.gov.lv/zalu-registrs/	Prices include VAT.
Morocco	Lower-middle	Direction des Médicaments et de la Pharmacie	https://dmp.sante.gov.ma/recherche-medicaments?search=	Prix fabricant hors taxe (PFHT; manufacturer price without tax) used.

© 2024 Barber MJ et al. *JAMA Network Open.*

Philippines	Lower-middle	Government maximum retail price, drug price reference index, government pharmacy price comparison facility 'Drug Price Watch'	https://dpri.doh.gov.ph/download , https://pharma.doh.gov.ph/maximum-drug-retail-price/ , https://dpw.doh.gov.ph	The lowest price across the three sources was used. For the Drug Price Watch search tool, which requires selection of a location, a 10km radius around Manila airport assumed. Some formulations in that database do not state a volume so were excluded.
South Africa	Upper-middle	South African Medicine Price Registry, Single Exit Prices	http://mpr.gov.za/PublishedDocuments.aspx#DocCatId=21	Medicine Price Registry (state purchase) used where available, Single Exit Prices (private market) used in other cases
United Kingdom	High	British National Formulary, Drug Tariff	https://bnf.nice.org.uk , https://www.drugtariff.nhsbsa.nhs.uk/	Lower price between the two sources used.
USA (NADAC)	High	CMS	https://data.medicaid.gov/nadac	National Average Drug Acquisition Cost (NADAC), published by the federal government.

*World Bank income classifications for 2021-22 are available from https://datahelpdesk.worldbank.org/knowledgebase/articles/906519-world-bank-country-and-lending-groups

© 2024 Barber MJ et al. *JAMA Network Open.*

eResults. Detailed Results

eTable 7. Sustainable costs for diabetes medicines, by formulation.

Medicine	Formulation*	Cost of production per unit (min, max)		Cost-based estimated generic/biosimilar price per unit (min, max)		Current lowest market price per unit (min, max)		% difference, comparing cost-based estimated prices to current lowest market prices
human insulin	vial	1.41	2.43	1.58	3.96	1.29	132.60	-97% to +22%
human insulin	cartridge	0.53	1.12	0.60	1.83	2.12	10.65	-83% to -72%
human insulin	disposable pen	0.83	3.62	0.94	5.89	1.87	6.35	-50% to -7%
NPH insulin	vial	1.42	2.45	1.60	3.98	1.29	132.10	-97% to +24%
NPH insulin	cartridge	0.54	1.13	0.60	1.83	2.14	6.14	-72% to -70%
NPH insulin	disposable pen	0.84	3.63	0.94	5.90	1.87	50.28	-88% to -50%
NPH insulin 30/70	vial	1.42	2.45	1.59	3.98	1.29	132.60	-97% to +23%
NPH insulin 30/70	cartridge	0.54	1.13	0.60	1.83	2.13	42.93	-96% to -72%
NPH insulin 30/70	disposable pen	0.84	3.63	0.94	5.89	2.00	90.69	-94% to -53%
insulin aspart	vial	2.88	4.35	3.24	7.06	4.34	138.90	-95% to -25%
insulin aspart	cartridge	0.96	1.68	1.08	2.72	2.79	171.10	-98% to -61%
insulin aspart	disposable pen	1.26	4.18	1.42	6.78	5.10	53.64	-87% to -72%
insulin lispro	vial	2.88	4.36	3.25	7.08	16.79	79.10	-91% to -81%
insulin lispro	cartridge	0.96	1.68	1.08	2.73	5.01	97.71	-97% to -78%
insulin lispro (100u/mL)	disposable pen	1.26	4.18	1.42	6.79	5.34	30.54	-78% to -73%
insulin lispro (200u/mL)	disposable pen (600 units)	1.94	5.06	2.18	8.22	16.26	17.11	-87% to -52%
insulin glulisine	vial	2.84	4.30	3.19	6.98	14.31	271.80	-97% to -78%
insulin glulisine	cartridge	0.95	1.66	1.07	2.70	4.25	10.13	-75% to -73%
insulin glulisine	disposable pen	1.25	4.16	1.40	6.76	4.70	105.39	-94% to -70%
insulin glargine (100u/mL)	vial	2.52	3.88	2.84	6.31	5.79	95.10	-93% to -51%
insulin glargine (100u/mL)	cartridge	0.86	1.54	0.96	2.50	5.49	13.05	-83% to -81%
insulin glargine (100u/mL)	disposable pen	1.16	4.94	1.30	6.57	2.98	28.41	-77% to -56%
insulin glargine (300u/mL)	cartridge (450 units)	1.12	1.88	1.26	3.06	14.75	14.75	-91% to -79%
insulin glargine (300u/mL)	disposable pen (450 units)	1.42	4.38	1.60	7.12	18.56	82.93	-91% to -91%
insulin detemir	vial	10.06	13.67	11.31	22.21	295.50	295.50	-96% to -92%
insulin detemir	cartridge	3.03	4.37	3.41	7.10	5.49	20.66	-66% to -38%
insulin detemir	disposable pen	3.33	6.87	3.75	11.16	9.80	88.74	-87% to -62%
insulin degludec (100u/mL)	vial	3.06	4.58	3.44	7.45	–	–	–

© 2024 Barber MJ et al. *JAMA Network Open.*

Medicine	Formulation*	Cost of production per unit (min, max)		Cost-based estimated generic/biosimilar price per unit (min, max)		Current lowest market price per unit (min, max)		% difference, comparing cost-based estimated prices to current lowest market prices
insulin degludec (100u/mL)	cartridge	1.01	1.74	1.14	2.83	10.24	19.71	-89% to -86%
insulin degludec (100u/mL)	disposable pen	1.31	4.24	1.48	6.89	11.27	97.65	-93% to -87%
insulin degludec (200u/mL)	disposable pen (600 units)	2.02	5.16	2.27	8.39	20.41	195.27	-96% to -89%
canagliflozin	100mg tablet	0.38	0.49	0.42	0.79	0.39	1.97	-60% to +8%
	300mg tablet	1.11	1.44	1.25	2.34	0.89	18.23	-87% to +40%
dapagliflozin	5mg tablet	0.02	0.03	0.03	0.05	0.10	2.26	-98% to -70%
	10mg tablet	0.04	0.05	0.04	0.08	0.13	17.56	-99.5% to -69%
empagliflozin	10mg tablet	0.04	0.05	0.04	0.08	0.14	2.09	-96% to -71%
	25mg tablet	0.08	0.10	0.09	0.16	0.29	18.24	-99% to -69%
dulaglutide	0.75mg pen	1.41	4.25	1.59	6.90	14.66	46.06	-89% to -85%
dulaglutide	1.5mg pen	2.3	5.4	2.59	8.78	17.89	47.01	-86% to -81%
dulaglutide	3mg pen	4.09	7.72	4.60	12.55	22.13	22.13	-79% to -43%
dulaglutide	4.5mg pen	5.87	10.04	6.61	16.31	20.81	213.05	-92% to -68%
exenatide (twice-daily)	5mcg /dose pen (60 doses)	0.75	3.49	0.85	5.67	98.98	770.21	-99% to -99%
exenatide (twice-daily)	10mcg /dose pen (60 doses)	0.88	3.66	0.99	5.94	78.34	770.21	-99% to -99%
liraglutide	18mg pen	7.66	12.39	8.62	20.13	31.42	340.56	-94% to -73%
semaglutide (injectable)	2mg pen	0.81	3.39	0.95	5.50	82.59	427.70	-99% to -99%
semaglutide (injectable)	4mg pen	0.96	3.53	1.08	5.74	88.54	861.03	-99% to -99%
semaglutide (oral)	3mg tablet	0.93	1.21	1.04	1.96	3.16	4.97	-67% to -61%
semaglutide (oral)	7mg tablet	1.15	1.49	1.29	2.42	3.16	9.65	-75% to -59%
semaglutide (oral)	14mg tablet	1.53	1.98	1.72	3.22	3.16	28.58	-89% to -46%

All figures in US$.
*Vials containing 1000 units in 10mL, disposable pens and cartridges containing 300 units in 3mL, unless otherwise indicated.
The ‘current lowest market prices’ represents the range of lowest available prices across the countries sampled, for those countries where price data were available.
Percentage difference between current lowest market prices and cost-based estimated generic prices represents the range from the difference between the lowest cost-based estimated generic price and the country with the lowest current market price, to the difference between the highest cost-based estimated generic price and the country with the highest current market price.

© 2024 Barber MJ et al. *JAMA Network Open.*

eFigure 2. Lowest market prices and cost-based prices (per month, US$): SGLT2 inhibitors

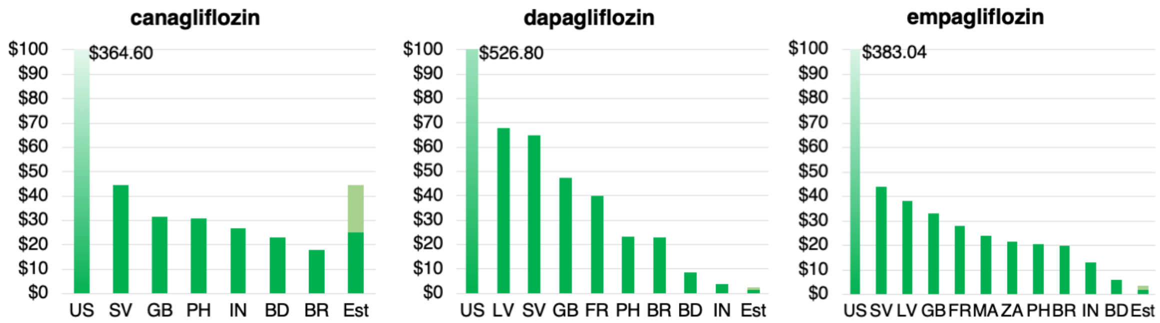

© 2024 Barber MJ et al. *JAMA Network Open.*

eFigure 3. Lowest market prices and cost-based prices (per month, US$): GLP1 agonists

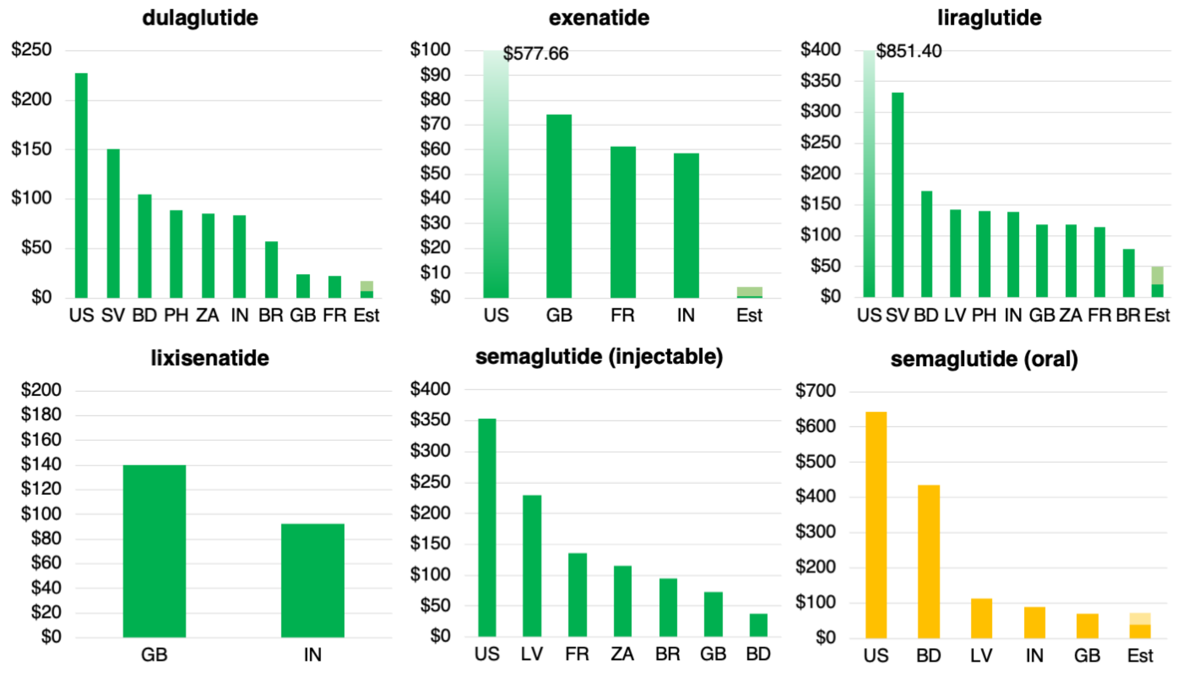

© 2024 Barber MJ et al. *JAMA Network Open.*

eFigure 4. Lowest market prices and cost-based prices (per month, US$): human insulins

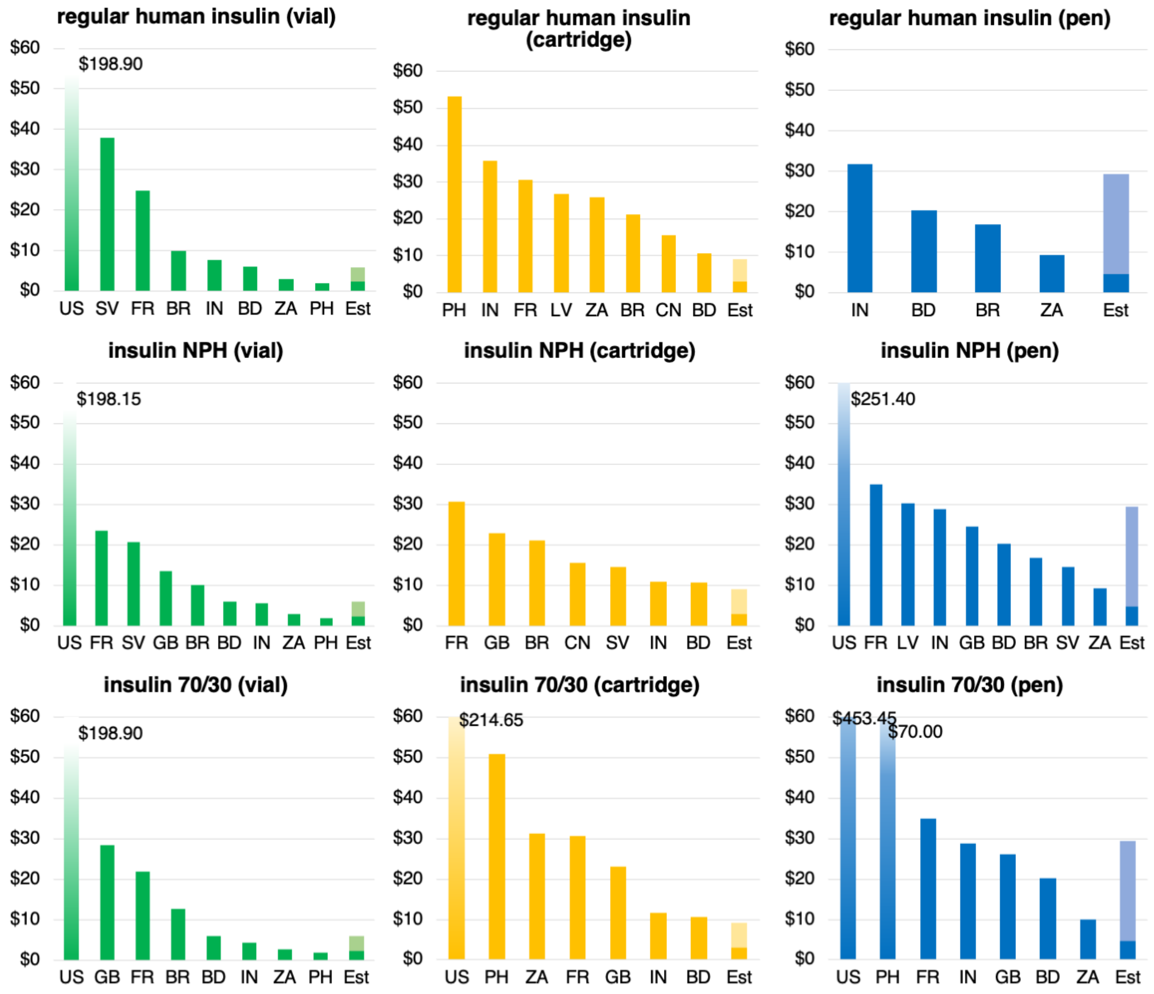

© 2024 Barber MJ et al. *JAMA Network Open.*

eFigure 5. Lowest market prices and cost-based prices (per month, US$): insulin analogues

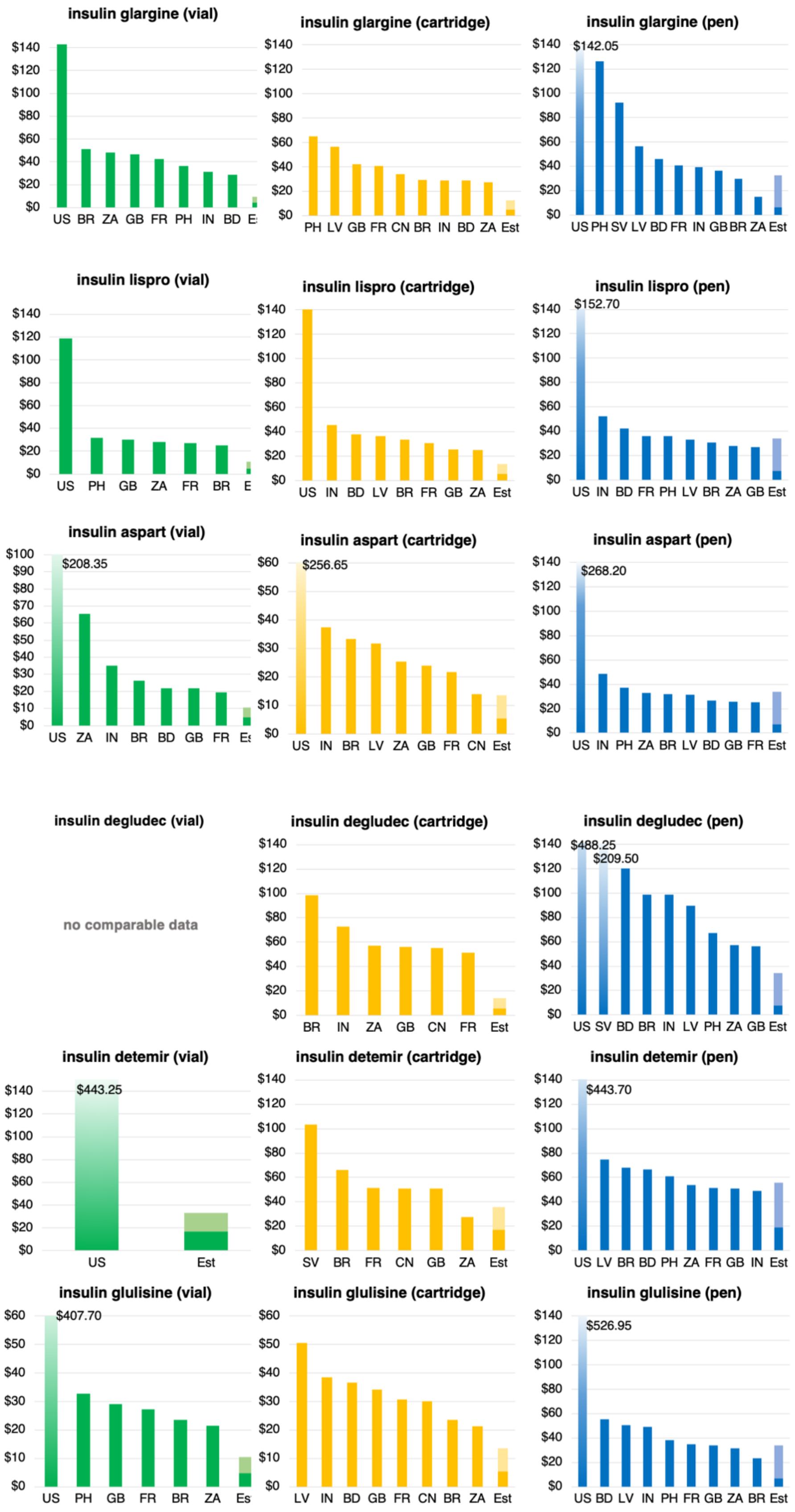

© 2024 Barber MJ et al. *JAMA Network Open.*

eReferences

1 IQVIA. Understanding Insulin Market Dynamics in Low- and Middle-Income Countries: Producers, supply and costs. 2021; published online Aug 26. https://www.iqvia.com/insights/the-iqvia-institute/reports/understanding-insulin-market-dynamics-in-low-and-middle-income-countries.

2 Chance RE, Glazer NB, Wishner KL. Manufacturing Process for Insulin Lispro. In: Walsh G, Murphy B, eds. Biopharmaceuticals, an industrial perspective. Dordrecht: Springer-Science+Business Media, B.V., 1999.

3 Sandow J, Landgraf W, Becker R, Seipke G. Equivalent Recombinant Human Insulin Preparations and their Place in Therapy. *European Endocrinology* 2015; **11**: 10.

4 Sreenivas S, Krishnaiah SM, Govindappa N, *et al.* Enhancement in production of recombinant two-chain Insulin Glargine by over-expression of Kex2 protease in Pichia pastoris. *Applied Microbiology and Biotechnology* 2015; **99**: 327–36.

5 Zimmerman RE, Stokell DJ, Akers MP. Aspart proinsulin compositions and methods of producing aspart insulin analogs therefrom. 2012.

6 European Medicines Agency. Apidra, INN-insulin glulisine. http://www.ema.europa.eu/docs/en_GB/document_library/EPAR_-_Scientific_Discussion/human/000557/WC500025246.pdf (accessed June 11, 2017).

7 European Medicines Agency. Tresiba : EPAR - Public assessment report. http://www.ema.europa.eu/docs/en_GB/document_library/EPAR_-_Public_assessment_report/human/002498/WC500139010.pdf (accessed June 11, 2017).

8 Edupuganti BR, Jagirdar H, Kumar M, Parthipan J, Yadav V, Sahib MK. Processes for refolding of insulin. 2011.

9 Coleman MP, Ortigosa AD, Sleevi MC, Chow K. Process for purifying insulin and analogues thereof. 2014.

10 Baeshen NA, Baeshen MN, Sheikh A, *et al.* Cell factories for insulin production. *Microbial Cell Factories* 2014; **13**. DOI:10.1186/s12934-014-0141-0.

11 Siew YY, Zhang W. Downstream processing of recombinant human insulin and its analogues production from E. coli inclusion bodies. *Bioresour Bioprocess* 2021; **8**: 65.

12 Petrides D, Sapidou E, Calandranis J. Computer-aided process analysis and economic evaluation for biosynthetic human insulin production—A case study. *Biotechnol Bioeng* 1995; **48**: 529–41.

13 Abdullah Baaj, Kenneth I Kaitin, Mari Serebrov. Manufacturing Strategy for Diverse Biologic Pipelines of the Future. Tufts Center for the Study of Drug Development. 2017; published online Oct. https://www.fdanews.com/ext/resources/files/2017/11/11-20-17-Tufts.pdf?1511557822.

14 Clendinen C, Zhang Y, Warburton RN, Light DW. Manufacturing costs of HPV vaccines for developing countries. *Vaccine* 2016; **34**: 5984–9.

© 2024 Barber MJ et al. *JAMA Network Open.*

15 United States Pharmacopeia. 1151. Pharmaceutical dosage forms. In: USP29. .

16 Sacha G, Rogers JA, Miller RL. Pre-filled syringes: a review of the history, manufacturing and challenges. *Pharmaceutical Development and Technology* 2015; **20**: 1–11.

17 Hill A, Barber MJ, Gotham D. Estimated costs of production and potential prices for the WHO Essential Medicines List. *BMJ Global Health* 2018; **0**: e000571.

18 Tax Foundation. Corporate Tax Rates around the World, 2022. 2022; published online Dec 13. https://taxfoundation.org/corporate-tax-rates-by-country-2022/.

19 Nxtgen. World Diabetes Injection Pens Market Research Report 2024. 2019 https://www.nxtgenreports.com/market-research-reports/world-diabetes-injection-pens-market-research.

20 Technavio. Research Report: Diabetic Pens Market (2020-2024): Increasing Prevalence of Diabetes to Boost Market Growth. 2020 https://www.technavio.com/report/diabetic-pens-market-industry-analysis.

21 Coalition for Epidemic Preparedness Innovations, Gavi, World Health Organization. COVID-19 Vaccine Manufacturing Presentation for DP Workshop. 2020; published online July 27. https://media.tghn.org/medialibrary/2020/11/20200727_DP_Workshop_presentation.pdf.

22 WHO Collaborating Centre for Drug Statistics. ATC/DDD Index 2022. 2022. https://www.whocc.no/atc_ddd_index/.

23 MSF Access Campaign, Santé Diabète. Towards Insulin for All: Operationalising the WHA74 Resolution on Diabetes. 2022; published online May. https://msfaccess.org/sites/default/files/2022-05/Diabetes_NCDs_TechBrief_AC-SD_Diabetes-resolution_ENG_May2022.pdf.

24 Gotham D, Barber MJ, Hill A. Production costs and potential prices for biosimilars of human insulin and insulin analogues. *BMJ Global Health* 2018; **3**: e000850.

25 Morin S, Segafredo G, Piccolis M, *et al.* Expanding access to biotherapeutics in low-income and middle-income countries through public health non-exclusive voluntary intellectual property licensing: considerations, requirements, and opportunities. *The Lancet Global Health* 2023; **11**: e145–54.

26 Tonghua Dongbao Pharmaceutical Co., Ltd. Annual Report. China, 2019.

27 Basu S, Yudkin JS, Kehlenbrink S, *et al.* Estimation of global insulin use for type 2 diabetes, 2018–30: a microsimulation analysis. *The Lancet Diabetes & Endocrinology* 2019; **7**: 25–33.

28 World Health Organization. Obesity and overweight. 2021; published online June 9. https://www.who.int/news-room/fact-sheets/detail/obesity-and-overweight.

29 Aylin Sertkaya, Andreas Lord, Clara Berger. Cost of generic drug development and approval. Eastern Research Group. 2021; published online Dec 31.

© 2024 Barber MJ et al. *JAMA Network Open.*

https://aspe.hhs.gov/sites/default/files/documents/20e14b66420440b9e726c61d281cc5a5/cost-of-generic-drugs-erg.pdf.

30 Anderson SL, Beutel TR, Trujillo JM. Oral semaglutide in type 2 diabetes. *Journal of Diabetes and its Complications* 2020; **34**: 107520.

31 European Medicines Agency Committee for Medicinal Products for Human Use. Assessment report: Rybelsus. 2020; published online Jan 30. https://www.ema.europa.eu/en/documents/assessment-report/rybelsus-epar-public-assessment-report_en.pdf.

32 Li T, Chandrashekar A, Beig A, *et al.* Characterization of attributes and in vitro performance of exenatide-loaded PLGA long-acting release microspheres. *European Journal of Pharmaceutics and Biopharmaceutics* 2021; **158**: 401–9.

33 Niazi S. Handbook of pharmaceutical manufacturing formulations, 2nd ed. New York: Informa Healthcare, 2009.

© 2024 Barber MJ et al. *JAMA Network Open.*
