# Supplementary material for: Estimated Sustainable Cost-Based Prices for Diabetes Medicines
Source: JAMA Netw Open. 2024 Mar 27;7(3):e243474. doi: 10.1001/jamanetworkopen.2024.3474 (PMC10973901; doi:10.1001/jamanetworkopen.2024.3474)
Supplement: Supplement 2. — Data Sharing Statement [file jamanetwopen-e243474-s002.pdf]

Data Sharing Statement

Barber. Estimated Sustainable Cost-Based Prices for Diabetes Medicines. *JAMA Netw Open*. Published March 25, 2024. doi:10.1001/jamanetworkopen.2024.3474

Data
Data available: Yes
Data types: Data (not involving human participants)
How to access data: Data can be accessed by emailing melissa.barber@yale.edu. We are not able to share the dataset on a website to comply with contract terms with the commercial database.
When available: With publication

Supporting Documents
Document types: None

Additional Information
Who can access the data: The data is available to anyone requesting the data.
Types of analyses: For any purpose
Mechanisms of data availability: With signed data access agreement (that the data will not be posted publicly, again, to comply with the commercial database's terms).
